# Leaf Temperatures in an Indian Tropical Forest Exceed Physiological Limits but Durations of Exposures Are Currently Not Sufficient to Cause Lasting Damage

**DOI:** 10.1111/gcb.70069

**Published:** 2025-02-10

**Authors:** Akhil Javad, Vikhyath Premugh, Rakesh Tiwari, Peddiraju Bandaru, Ron Sunny, Balachandra Hegde, Santiago Clerici, David Galbraith, Manuel Gloor, Deepak Barua

**Affiliations:** ^1^ Department of Biology Indian Institute of Science Education and Research Pune Maharashtra India; ^2^ School of Geography University of Leeds Leeds UK; ^3^ Sahyadri Wildlife and Forest Conservation Trust Sirsi India

**Keywords:** agroforestry, climate change, leaf temperature, photosynthesis, thermotolerance, tropical forests

## Abstract

Increasing temperatures in the tropics will reduce performance of trees and agroforestry species and may lead to lasting damage and leaf death. One criterion to determine future forest resilience is to evaluate damage caused by temperature on Photosystem‐II (PSII), a particularly sensitive component of photosynthesis. The temperature at which 50% of PSII function is lost (*T*_*50*_) is a widely used measure of irreversible damage to leaves. To assess vulnerability to high temperatures, studies have measured *T*_*50*_ or leaf temperatures, but rarely both. Further, because extant leaf temperature records are short, duration of exposure above thresholds like *T*_*50*_ has not been considered. Finally, these studies do not directly assess the effect of threshold exceedance on leaves. To understand how often, and how long, leaf temperatures exceed critical thresholds, we measured leaf temperatures of forest and agroforestry species in a tropical forest in the Western Ghats of India where air temperatures are high. We quantified species‐specific physiological thresholds and assessed leaf damage after high‐temperature exposure. We found that leaf temperatures already exceed *T*_*50*_. However, continuous exposure durations above critical thresholds are very skewed with most events lasting for much less than 30 min. As *T*_*50*_ was measured after a 30‐min exposure, our results suggest that threshold exceedances and exposure durations for lasting damage are currently not reached and will rarely be reached if maximum air temperatures increase by 4°C. Consistent with this, we found only minor indications of heat damage in the forest species. However, there were indications of heat‐induced reduction in PSII function and damage in the agroforestry leaves which have lower *T*_*50*_. Our findings suggest that, for forest species, while high‐temperature thresholds may be surpassed, durations of exposure above thresholds remain short, and therefore, are unlikely to lead to irreversible damage and leaf death, even under 4°C warming.

## Graphical abstract

Lethal damage of forest canopy leaves depends on two factors—exposure above physiologically unsustainable temperatures and duration of such exposure. We show that for a tropical forest from the Western Ghats range in the Indian Peninsula, leaf temperatures are already reaching the threshold temperatures, but the durations of exposure are too short to cause lasting damage.

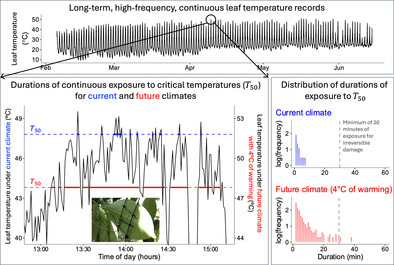

## 1 Introduction

Global mean surface temperature is expected to increase by 2°C above temperatures in 1750 at the peak of global warming (Hansen et al. 2022). Temperatures on land are predicted to rise at approximately twice the rate as compared to sea surface temperatures (Byrne and Gorman 2018), and therefore, are expected to increase by as much as 3.6°C by the same time. Additionally, these increases in mean temperatures will be accompanied by increased frequencies of exposure to extreme temperatures (e.g., Fischer, Beyerle, and Knutti 2013). All life forms on earth, including plants will be subjected to these increases, and the consequences of exposure to increased intensity and frequency of extreme temperatures could be severe (e.g., Deutsch et al. 2008; Slot and Winter 2016). However, it is not clear how plants, including forest trees and agriculturally important crops, will be affected by these higher temperatures.

Tropical plants may be particularly vulnerable to future climates with increased mean temperatures and more frequent exposure to extreme temperatures. Tropical plants may possibly already be living close to their upper limits of temperature tolerance (Doughty and Goulden 2008; Doughty et al. 2023; O'Sullivan et al. 2017). Additionally, at a fundamental biochemical level, the sensitivity of many vital processes to temperature are similar and thus are not expected to vary much with latitude (Björkman and Demmig 1987; Slot et al. 2021). As temperatures in the tropics are among the highest, limits of functioning of plants because of increases in temperatures will most likely be reached there (Deutsch et al. 2008). It is therefore expected that negative effects of increased temperature on vegetation and specifically forests will be particularly strong in these regions. Consequences of increasing temperatures in the tropics may also differ from the extra‐tropics because there is no vegetation type which can move in from lower latitudes to replace vegetation shifting away from the tropics into more moderate climates. Potential loss of tropical forest species in these regions would thus be irreversible on time‐scales relevant to humans with possible consequences at various levels.

The temperature sensitivity and limit of photosynthesis is one key determinant of how tropical trees and plants will fare in a warmer world. Experiments have shown that among the components of the photosynthetic apparatus, Photosystem II is particularly vulnerable to high temperature (Berry and Björkman 1980; Havaux, Greppin, and Strasser 1991), the first of the two photosystems operating in series. Specifically, the ratio of relative difference of fluorescence of a leaf exposed to a saturating light pulse (*F*_*m*_) and very low light (*F*_*0*_), (*F*_*m*_ *−F*_*0*_)/*Fm* (or *F*_*v*_/*F*_*m*_ with *F*_*v*_ defined as *F*_*v*_ *= F*_*m*_−*F*_*0*_) has been shown to be equal to the maximum quantum yield of photosynthesis (mol CO_2_ fixed per mol photons captured) (Butler and Kitajima 1975; Schreiber and Berry 1977; Björkman and Demmig 1987). Furthermore, it has been shown that deviations from its maximum value are an indicator of stress, and levels approaching 50% of its maximum level, an indicator of irreversible damage to Photosystem II (Berry and Björkman 1980; Schreiber and Berry 1977; Bilger, Schreiber, and Lange 1984). Temperature that reduces the ratio to 50% of its maximum, *T*_*50*_, is typically measured on leaves which have been exposed for a fixed duration of time to a fixed temperature in the dark. It is a measure of a cumulated, not of an instantaneous, heat effect. Ignoring the cumulative nature of this measure when interpreting exceedance of leaf temperature above *T*_*50*_ may lead to erroneous conclusions regarding lethality (e.g., Neuner and Buchner 2023). Besides high temperatures causing damage to Photosystem II, they also limit photosynthesis if they exceed the upper limit *T*_*max*_ above which photosynthetic rate is zero.

Leaf temperatures are the result of a balance between absorbed solar radiation (direct and reflected, e.g., off other leaves), and sky and ground thermal radiation which heat the leaf, and thermal radiative and evaporative heat losses which cool the leaf. An additional process, sensible heat gain or loss, can either heat or cool the leaf depending on leaf‐to‐air temperature difference. Because of the small leaf thermal mass, temperature adjustment time to changes in energy fluxes are rapid (~10 s) and, provided soil water is not limited, a plant may actively counteract warming by evaporative cooling (e.g., Jones 2014). Overall, while the different leaf temperature determinants are well understood, in principle, the resulting maximum leaf temperatures experienced in the tropics are not well understood, and its effect on leaf damage uncertain.

There are several reasons. There are only a few existing single leaf temperature records in the tropics (3.02° S 54.97° W, 20.4°C–31.7°C, Doughty and Goulden 2008; 23.32° S 45.09° W, 17°C (mean air temperature), 3°C–20°C, Fauset et al. 2018; 14.64° S 52.37° W, 15°C–45°C, Araújo et al. 2021; 21 to 27° N 101° E, 28.1°C–45.9°C, Kitudom et al. 2022; 2.11° S 30.85° E, 24.3°C (mean air temperature), 35.2°C (maximum air temperature), Manzi et al. 2024) of which, to our knowledge, only one is in the warmer regions of the tropics with air temperatures reaching and sometimes exceeding 40°C. Existing records tend to cover only a short period and/or are sampled with coarse time resolution. Thus, exposure time statistics are difficult to determine. In contrast to the sparseness of suitable leaf temperature records, data of measures of thermal limits to photosystems (*T*_*50*_) and more generally photosynthesis of tropical forest trees are becoming increasingly available (Sastry and Barua 2017; Sastry, Guha, and Barua 2018; Perez and Feeley 2020; Slot et al. 2021; Araújo et al. 2021; Kitudom et al. 2022; Tarvainen et al. 2022; Tiwari et al. 2021; Kullberg et al. 2024). Several studies have focused on both thermotolerance and “thermal safety margins,” the difference between leaf temperatures and measures of thermal limits (like *T*_*50*_), in the tropics (e.g., Araújo et al. 2021; Doughty and Goulden 2008; Doughty et al. 2023; Kitudom et al. 2022); however, they have generally not considered the role of exposure time above temperature thresholds to assess leaf vulnerability to heat. They also did not determine in situ whether there is leaf damage in some form after excess heat spells with leaf temperatures exceeding thermal thresholds.

Here we report on long‐term (up to 4.5 months) continuous high frequency (1 min^−1^) tropical forest leaf temperature measurements in an already warm tropical region aiming to expand our knowledge about leaf temperatures reached, their relation to *T*_*50*_ and *T*_*max*_, statistics of durations of exposure above thresholds, and the interplay of the two on leaf health. Specifically, we report on comparisons of leaf temperature data of four dominant forest species (three evergreen and one deciduous) and 13 agroforestry species with species specific *T*_*50*_, *T*_*5*_ (the leaf temperature at which *F*_*V*_/*F*_*m*_ is reduced by 5% from its maximum level) and *T*_*max*_, at a site close to Sirsi, Karnataka in the Western Ghats during the dry and hot season of 2023. Agroforestry systems are common accounting for more than 10% of the landuse in the region (Rizvi et al. 2020). They are traditionally used in this area where perennial woody species are used to complement crops and livestock rearing. The selected agroforestry species (Table S1) are commonly cultivated in the region and are important for the local economy.

Our long‐term records permit us also to determine the statistics of how long leaves experience temperatures above the threshold measures. Sirsi experiences a climate representative for Western Ghats, India. Air temperatures during the dry season at the site are at the high end of the tropics (up to ~40°C). Similarly to most places in the world, daily maximum temperatures have been increasing over the past decades (Mann and Gupta 2022).

To assess, to a limited extent, the predictive power of exposure above threshold measures, we furthermore measured dark‐adapted, maximum quantum yield (*F*_*v*_/*F*_*m*_) of top‐of‐canopy sun‐exposed leaves measured towards the end of the dry season as well as for shaded (or partially shaded) leaves. As a second diagnostic, we report on visually recognizable damage statistics for leaves with differing exposure to direct sunlight both for forest and agroforestry species. Based on our data, we then evaluate the danger of limits to leaf photosynthesis caused by high‐temperature exposure, currently and in the future, for this already very warm site and put the results in perspective with the conclusions of recent studies about future heat related vulnerability of tropical forests.

## 2 Methods

### 2.1 Site and Species Description

The study was conducted in forests near Hosagadde village close to Sirsi, Karnataka (14°28′ 44′′ N, 74°45′ 30′′ E) at an elevation of approximately 520 m above sea level (Figure S1). The site is located on the Western Ghats, a mountain range that runs parallel to the West coast of peninsular India. The climate is monsoonal and most of the annual average rainfall of around 3000 mm falls between June and October (climate data from Climate Research Unit, CRU TS v4.08, Harris et al. 2020). The period between November and May represents the dry season with negligible precipitation (178 mm). Air temperatures increase after the withdrawal of the monsoons in October and are the highest in April during the latter half of the dry season (Figure S1). This period also corresponds to the times with the highest solar radiation (a daily peak of 1902.5 μmol photons m^−2^ s^−1^ on average during the measurement period) and the driest soil conditions (soil moisture content of 0.179 m^3^ m^−3^ between March and May).

The vegetation in this region consists of tropical evergreen and semi‐evergreen forests in the western parts which transition to moist deciduous forests in the eastern parts of the region with lower annual rainfall. We selected three evergreen species (*Psydrax dicoccos* Gaertn., *Memecylon umbellatum* Burm.f. and *Olea dioica* Roxb.), one deciduous species (*Terminalia paniculata* B.Heyne ex Roth), and 13 agroforestry species (Table S1) for this study. The three evergreen species are abundant in the evergreen forests in this region, while the deciduous species (*Terminalia paniculata*) is the most dominant deciduous species in the semi‐evergreen and the moist deciduous forests. Canopy cover in the forest edges where our focal individuals of the four forest tree species were present ranged from approximately 50% to 100%, whereas the canopy cover in the plantations where our agroforestry species were present was approximately 25%–75%. The 13 agroforestry species include natives of the region, and non‐natives, introduced to the region over the past few centuries, from wet or seasonally dry parts of the tropics (Table S1).

### 2.2 Leaf Temperature Measurements

Continuous leaf temperature measurements were made on randomly chosen, mature, upper‐canopy, sun‐exposed leaves of the four forest species while leaf temperatures of agroforestry species, which were accessible using ladders, were measured using a handheld thermal camera (Javad et al. 2025). The continuous measurements were made for three to four leaves from two (for one species) to four individuals between February and June 2023 using leaf temperature sensors (LAT‐B2, Ecomatik, Munich, Germany) with a sampling frequency of 1 min^−1^. The start date of the measurements differed for individuals and species due to availability of mature leaves. Three of the species, *Psydrax*, *Olea*, and *Memecylon*, are evergreen and maintain leaves throughout the year. *Terminalia* is leafless during most of the dry season and flushes new leaves around the middle of April. Therefore, for *Terminalia*, the measurements were only initiated at the end of April. Although the leaves of *Terminalia* avoid the hottest temperatures during March and April, the leaves are still exposed to considerably hot temperatures of May. Each sensor had a pair of thermistors that were installed underneath each leaf, one touching the leaf surface, and the other, tilted slightly downwards to measure air temperature adjacent to the leaf (Figure S2). Care was taken to ensure that both thermistors were directly under the leaf and not exposed to direct radiation. Before installing the thermistors on the leaves and at the end of the measurement period, all thermistors were exposed to identical temperatures to correct for differences between thermistors.

To understand the maximum daily leaf temperatures experienced by the agroforestry species (and the four forest species), we also measured instantaneous leaf temperatures for mature upper‐canopy, sun‐exposed leaves using a handheld thermal camera (FLIR C2, Teledyne FLIR, Wilsonville, Oregon, USA). Measurements were made daily at times of peak solar irradiation between 12 p.m. and 3 p.m over a two‐week period toward the end of the dry season (May 8–May 20, 2023) for six to ten leaves from six individuals of each species. A comparison of temperatures measured with thermal camera and thermistors revealed good agreement between the two (Figure S3).

### 2.3 Chlorophyll Fluorescence (*F_v_*/*F*_*m*_) Versus Temperature Curves and Determination of *T*_*50*_

We used a PAM 2500 fluorometer (Walz, Effeltrich, Germany) to measure the temperature response of dark‐adapted chlorophyll a fluorescence (*F*_*v*_/*F*_*m*_) which represents the maximum potential quantum yield of photosystem II (PSII). *F*_*v*_/*F*_*m*_ is calculated as (*F*_*m*_−*F*_*0*_)/*F*_*m*_, where *F*_*m*_ is the maximum fluorescence yield of dark‐adapted leaves exposed to a short pulse of high intensity light, and *F*_*0*_ is the basal fluorescence yield of dark‐adapted leaves under very low light (Baker 2008). *F*_*v*_/*F*_*m*_ is a measure of PSII integrity and function and is a physiological measure that is very sensitive to high temperature (Bilger, Schreiber, and Lange 1984; Krause et al. 2010). The temperature that results in a 50% reduction in PSII function (*T*_*50*_) is widely used as a measure of leaf high‐temperature tolerance (Doughty et al. 2023) and is closely related to temperatures that result in necrotic damage and leaf death (Bilger, Schreiber, and Lange 1984; Krause et al. 2010). The temperature that results in a 5% reduction, in PSII function (*T*_*5*_), represents a threshold for initial damage to the photosynthetic machinery (Curtis et al. 2014). Both physiological thresholds, *T*_*5*_ and *T*_*50*_, are measures of accumulated damage caused by continuous exposure to temperatures above thresholds for a period of time, here 30 min, and not a result of instantaneous exposure. Some studies use the term *T*_*crit*_ for *T*_*5*_ (Doughty et al. 2023). However, *T*_*c*_ (or *T*_*crit*_ or *T*_*critical*_) is historically used in dynamic assays measuring the breakpoint of basal fluorescence (*F*_*0*_) in response to increasing temperatures (Schreiber and Berry 1977). As *T*_*c*_ is determined differently to the methodology of *F*_*v*_/*F*_*m*_ and leaf exposure for 30 min at *T*_*c*_ leads to 50% dead leaf area (Schreiber and Berry 1977), the use of *T*_*c*_ or *T*_*crit*_ or *T*_*critical*_ for *T*_*5*_ is confusing and, hence, not used here.

Mature, upper‐canopy, sun‐exposed leaves from five to six individuals of the study species were collected in the evening and rehydrated with petioles immersed in a beaker of water in a sealed plastic bag overnight between September and December 2019. Leaf discs (~0.8 cm diameter) were excised from these leaves the next morning, placed between two layers of muslin cloth, covered with aluminum foil and put in a sealed plastic bag with moistened tissue paper at the bottom. These bags were subsequently immersed in a temperature‐controlled water bath (Julabo, Model F25, Seelbach, Germany) for 30 min. Previous studies and preliminary results suggest that a 30 min of exposure results in irreversible damage with limited recovery of PSII function (Curtis et al. 2014; Sastry, Guha, and Barua 2018). Some studies used 15 min of exposure instead of 30 min. However, in our experience, 15 min of exposure is a less reliable indicator of irreversible damage. Water baths were preset to temperatures required to attain the desired leaf temperatures (25°C, 40°C, 45°C, 47.5°C, 50°C, 52.5°C, and 55°C), and the leaf temperature was monitored with a fine gauge thermocouple attached to the underside of a leaf disc in the bag (dummy leaf and bag placed in the water bath that was not used for the assays). Preliminary trials were conducted to determine the water bath temperatures required to attain the desired leaf temperatures. After the heat treatment, leaf discs were stored in petri dishes with wet tissue for 24 h after which the discs were allowed to dark adapt at room temperature in a dark felt envelope for 30 min and chlorophyll a fluorescence (*F*_*v*_/*F*_*m*_) was measured with a PAM 2500 fluorometer.

The *F*_*v*_/*F*_*m*_‐temperature responses were used to fit a four‐parameter logistic sigmoid curve using the R package “*drc*” (Ritz et al. 2015). The lower asymptote was set to zero assuming a complete loss of PSII function at very high temperatures. The temperatures that resulted in a 5% (*T*_*5*_) and 50% (*T*_*50*_) decrease in PSII were estimated from these curves for each examined individual and species estimates calculated as the mean of these individuals (Figure S4A–B).

### 2.4 CO_2_ Assimilation Versus Temperature Curves

Assimilation versus temperature curves were measured for the four examined forest species only because of time and resource limitations. Measurements were made during March to mid‐April 2021 in situ on mature and healthy leaves on accessible, sun‐exposed branches (we pulled down the branches located 2–5 m from the ground and stabilized using ropes) using a LI‐6400XT with the LI‐6400‐40 leaf chamber (LI‐COR, Lincoln, Nebraska, USA). Irradiance levels were set to 1000 μ mol photons m^−2^  s^−1^ (10% red; far red off), CO_2_ levels to 400 μ mol CO_2_ mol^−1^, and relative humidity to 55% ± 15%. The selected leaves were allowed to stabilize in the closed leaf chambers for at least 10 min at 20°C. Net photosynthetic rates were subsequently measured at 20°C, 25°C, 30°C, 35°C, 40°C, and 45°C. Photosynthetic rates were allowed to stabilize at each temperature step before the recordings were made. *T*_*opt*_, the temperature at which the CO_2_ assimilation rate is maximal, and *T*_*max*_, the upper temperature limit at which photosynthesis rate is positive, was estimated following (Docherty et al. 2023) (Table S2).

### 2.5 Leaf Heat Damage Assessment

To understand the effects of exposure to high temperatures on leaves, we measured and compared *F*_*v*_/*F*_*m*_ of sun‐exposed and shaded leaves of the four forest species and for a subset of seven agroforestry species (Cinnamon, Clove, Cocoa, Coffee, Lemon, Pepper, and Vanilla) towards the end of the dry season (Javad et al. 2025). The number of agroforestry species for *F*_*v*_/*F*_*m*_ based damage assessment was reduced to seven due to time and personnel constraints. The seven species were chosen to be representative of the range of *T*_*50*_ values of the agroforestry species. Because shaded leaves experience lower light levels and, therefore, lower leaf temperatures compared to sun‐exposed leaves, we considered their values as a proxy of a “control.” If sun leaves have lower *F*_*v*_ */F*_*m*_ than shaded leaves, it indicates the effect of light and heat unless the leaves are completely dried out (Havaux, Greppin, and Strasser 1991). The measurements were made for six sun‐exposed and six shaded leaves, for three to four individuals of the forest species, and five to six individuals of the agroforestry species. Leaves were collected early in the morning and allowed to rehydrate for at least 2 h with their petioles immersed in a beaker of water while enclosed in a sealed plastic bag. Following rehydration, these leaves were allowed to dark adapt for at least 30 min before measurement of *F*_*v*_ */F*_*m*_.

In addition to the *F*_*v*_ */F*_*m*_ measurements, we also estimated the percentage of leaves with some visible, potentially heat‐induced damage or necrosis for the four forest species and the 13 agroforestry species (Figure S5). Sun‐exposed leaves of at least three individuals were randomly selected and the number of leaves with visible damage determined. A minimum of 10 and up to 80 leaves were sampled from each individual. Damage that could be attributed to pests or pathogens were not counted as heat related damage. We attribute leaf necrotic damage based on the following observations and criteria. Heat‐related leaf necrotic damage is visually uniform (dark) across a well localized region of a leaf as opposed to spot like damage caused by pathogens. Heat damage decreases from top‐of‐canopy sun exposed leaves to shaded leaves differently from damage due to dehydration (which is uniform) and due to pathogens.

### 2.6 Estimation of Leaf Exposure Times Above Thermal Thresholds for Current and Future Climates

The damaging effect of leaf temperature exceedance above thermal thresholds (*T*_*5*_, *T*_*50*_) depends on exposure time (e.g., Neuner and Buchner 2023). Our long‐term forest leaf temperature records sampled with frequency of one per min permits us, as a first step, to determine the duration of uninterrupted exposure each time a leaf exceeds a threshold temperature, and then determine the statistics of such continuous exposure durations during the 4.5 months of the measurement period. To provide an estimate of how these exposure times may change if air temperature increases by 2°C and 4°C, we follow a pragmatic approach. We repeat the procedure described above but for *T*_*5*_ and *T*_*50*_ reduced by 2°C and 4°C, respectively, which is equivalent to increasing air temperatures by 2°C and 4°C. The implicit assumptions are (i) that the relationship between *T*_*leaf*_—*T*_*air*_ and *T*_*air*_ stays the same, and (ii) there is no acclimation in threshold temperatures with warming. In addition, we also examined how acclimation would affect the statistics of durations of continuous exposure above threshold temperatures assuming *T*_*50*_ acclimates by a fixed rate of 0.38°C per 1°C rise in air temperatures (Rao et al. 2023; O'Sullivan et al. 2017).

### 2.7 Data Analysis

We used analysis of variance (ANOVA) to test for differences in *F*_*v*_/*F*_*m*_ across leaves that are either sun exposed or shaded with species as a nested variable. We also used ANOVA to test for *T*_*50*_ across forest versus agroforestry species and open versus understorey species. All the analyses were done using R 4.4.0 (R Core Team 2024) using packages “*multcompView*” (Graves, Piepho, and Dorai‐Raj 2024), and “*ggplot2*” (Wickham 2016).

## 3 Results

### 3.1 Inter‐Species Variation in Temperature Thresholds (*T_opt_*, *T*_*max*_, *T*_*5*_ , *T*_*50*_ )

Species‐specific estimates of *T*_*5*_ ranged from around 42°C to greater than 46°C, and for *T*_*50*_, from 46°C to around 51°C (Table S2). These estimates of the upper limits of heat tolerance were typically lower for the agroforestry species than for forest species (Tables S2 and S3). Both *T*_*5*_ and *T*_*50*_ were significantly different across species (Table S3). The optimum and the maximum temperatures for photosynthesis were quantified only for the four forest species and ranged from around 28°C–32°C for *T*_*opt*_, and from 43°C to 46°C for *T*_*max*_ (Table S2).

### 3.2 Continuous Leaf Temperature Measurements

**FIGURE 1:**
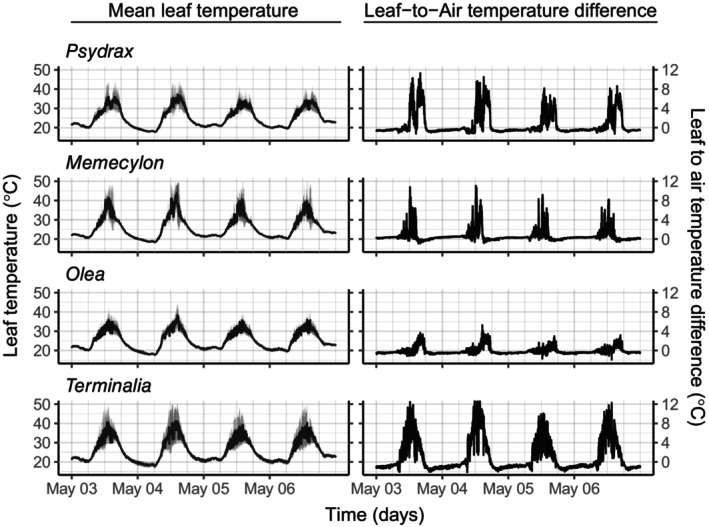
Mean leaf temperature records across leaves of the four forest species—*Memecylon*, *Psydrax*, *Olea*, and *Terminalia*—examined in the study for a representative 4‐day period (left column). Gray shade shows the variance across the three to four leaves of each species at a given time. Right column shows the corresponding leaf‐to‐air temperature differences across the same period for one of the leaves of each species.

**FIGURE 2:**
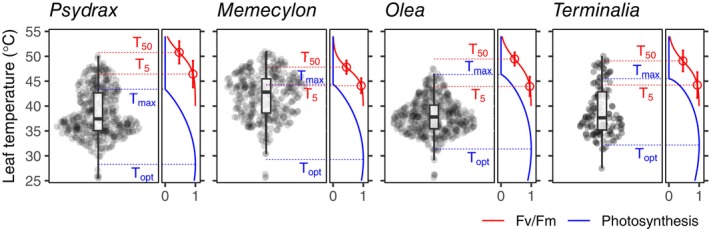
Daily maximum leaf temperatures of forest species measured continuously during the dry season (typically several months) using thermistors attached to leaves (Table 1 and Figure S2) and species specific *T*_*50*_, *T*_*5*_, *T*_*max*_ and *T*_*opt*_.

Leaf and air temperatures for the four forest species followed a strong diurnal cycle (Figure 1). During the night and for parts of the day when solar radiation levels were low, leaf and air temperatures were not different from each other (within approximately ± 0.5°C). In contrast, during times of peak solar radiation around mid‐day and early afternoon, leaf temperatures exceeded air temperatures substantially and were as much as 12°C higher than air temperature (Figures 1, S6, and S7). The maximum temperatures experienced by leaves differed between species and individuals (Figures 1 and 2, Table S4). During the day, leaf temperatures almost always exceeded air temperatures and were substantially higher than air temperatures for several hours (Figures 1, and S7). Distributions of the difference between leaf and air temperature were heavily skewed, with positive differences reaching substantially higher values than negative differences (Figure S7; Table S5).

### 3.3 Maximum Leaf Temperatures and Durations of Exposure Above Thermal Thresholds

Overall, the maximum leaf temperatures experienced by the leaves of the four forest species during the study period, and the exceedance of threshold measures vary between leaves of a given species, and across species, partially because of the differences of the values of these threshold measures mentioned before (Figure 2; Tables S2, and S5). Starting with *T*_*max*_, the maximum temperature above which carbon assimilation ceases, we found that the total time spent per day with *T*_*leaf*_ > *T*_*max*_ was very short. For *O. dioica* , leaf temperatures were mostly never higher than *T*_*max*_, and barring a few extreme leaf temperatures, the time spent above *T*_*max*_ was in the range of minutes. Assuming no other changes in the future except air temperature, the time leaves are exposed to temperatures greater than *T*_*max*_ will increase from a range of 0 to 9.6 min on average per day across the four forest species to 0.3 to 24.4 min under 2°C warming and 2.1–44.9 min under 4°C warming (Table S5B). On average for a 4°C warming scenario, exposure to *T*_*max*_ was < 40 min per day. Based on these results, the duration of time when carbon assimilation is not possible remains very low even under a 4°C warming scenario.

**FIGURE 3:**
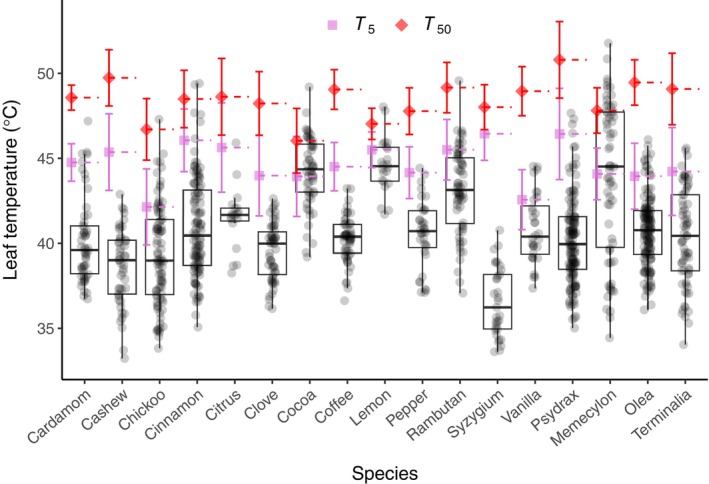
Leaf temperatures measured daily around mid‐day using a handheld thermal camera (FLIR C2) during a two‐week period in May 2023 in relation to species specific threshold measures *T*_*5*_ (purple) and *T*_*50*_ (red). Measurements were made on 13 agroforestry species and the four forest species investigated in this study (Table S1). Per species at least six sun exposed and shaded leaves from three to five individuals were probed.

**FIGURE 4:**
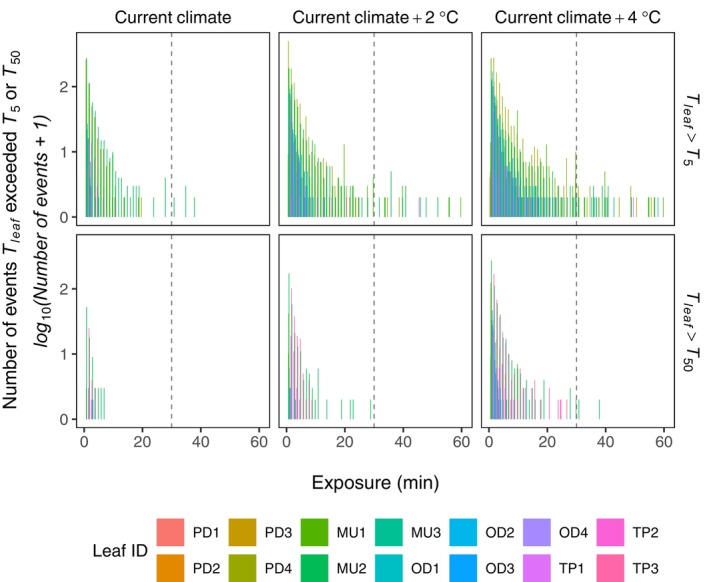
Frequency distribution of duration of uninterrupted leaf exposure above threshold temperature for two threshold measures (*T*_*5*_, *T*_*50*_) during the measurement period in 2023 and for two future scenarios of 2°C and 4°C air temperature increases relative to 2023.

For *T*_*50*_, the temperature threshold that results in irreversible damage, maximum temperatures of leaves of three of the forest tree species come regularly close to and occasionally exceed *T*_*50*_ (Figures 2 and 3; Tables S5, and S6). For none of the species and leaves for current climate (year 2023) that the duration of uninterrupted exposure above *T*_*50*_ is more than 10 min, and for most of them, the maximum exposure time is very brief (in the range of minutes) (Figure 4). For all the 14 measured leaves, the durations of above *T*_*50*_ temperature exposure are much below 30 min. For a future 2°C warming, uninterrupted exposure of *T*_*leaf*_ > *T*_*50*_ increases but remains below 30 min for all leaves. For a 4°C scenario, one out of our 14 leaves will be exposed to uninterrupted exposure by much more than 30 min. Among the 14 leaves, one leaf would thus, with certainty, be heavily damaged (possibly die) while heavy damage/death is unlikely for the other 13 leaves. If we take into account the acclimation of *T*_*50*_ with rise in air temperatures, our conclusions are further strengthened (Table S5C).

Considering the threshold measure *T*_*5*_, currently two out of 14 leaves are exposed to more than 30 min of uninterrupted exposure under current conditions, while it is much less for the other leaves. Under a 2°C warming scenario, the number of leaves exposed to more than 30 min above *T*_*5*_ increases to four leaves, and under 4°C warming, to six leaves (43% of the measured leaves).

Our dataset for agroforestry species is limited to spot measurements, thus, we do not have information about duration of uninterrupted exposure of leaf temperature above the measured physiological thresholds. Based on the spot measurements, we find that, for almost all species, a substantial percentage of the measured leaf temperatures exceeds *T*_*5*_, and for approximately half of the species, a small percentage exceeds *T*_*50*_.

### 3.4 In Situ Assessment of Heat Effects on Leaves

**FIGURE 5:**
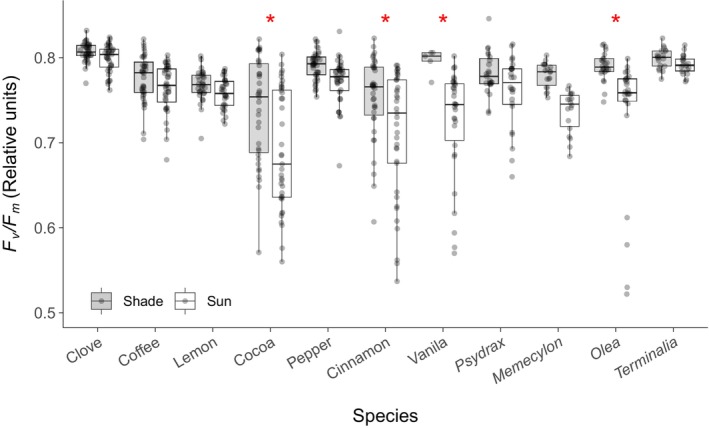
Dark‐adapted quantum efficiency *F*_*v*_/*F*_*m*_ measured on shade (gray) and sun exposed (white) leaves at end of dry season of four forest species and seven agroforestry species. Red asterisks denote species with significantly different sun and shade *F*_*v*_/*F*_*m*_ from a nested ANOVA across species and leaf types (shade vs. sun exposed).

Photosystem II quantum efficiency, a diagnostic of differences in performance/indicator of heat damage, of sun‐exposed versus shaded leaves, reveals that the quantum efficiencies of the sun‐exposed leaves, determined at the end of the measurement period, are slightly reduced, compared to the less sun exposed leaves. Nonetheless, the differences are only significant for four species and thus, only for these four species may indicate some negative heat effect (although affecting only a small part of the leaf) to the leaf's Photosystems II (Figure 5).

As a second diagnostic of leaf damage, we examined leaves visually (Figure S5) toward the end of the measurement period. Sun‐exposed leaves of several agroforestry species developed black areas on leaves, damage which we associate with excess heat dose based on previous examination of such patterns. We view this association as a preliminary attempt and, thus, as somewhat tentative (e.g., it is difficult to conclusively exclude a role played by pathogens or droughts). Leaves of two forest species also had some visible damage but no black areas. There is a correspondence between cumulated exposure time to high temperatures and percentage of leaves with necrotic damage for the forest species (Table 1) but no clear relationship between percentage of leaves, which have been exposed to temperatures above critical levels, and leaf damage, for the agroforestry species (Table S6).

**TABLE 1:** Summary of long‐term leaf temperature measurements of 4 forest species—*Psydrax*, *Memecylon*, *Olea*, *and Terminalia*—using thermistors. Total number of days the measurements were done for each individual tree of the four species, mean continuous exposures across the measurement period and the percentage of days when leaf temperatures were above the physiological thresholds, *T*_*max*_, *T*_*5*_, and *T*_*50*_, and percentages of leaves with some visible necrotic damage attributed to excess heat are presented.

Species/Leaf	Record length (days)	Mean exposure (min/day) *T*_*leaf*_ >			Percentage of days (%) *T*_*leaf*_ >			Necrotic damage (%)
		*T*_*max*_	*T*_*5*_	*T*_*50*_	*T*_*max*_	*T*_*5*_	*T*_*50*_	
*Psydrax*								
PD1	112	0.0	0.0	0.0	2.7	0.0	0.0	0.0
PD2	112	0.1	0.0	0.0	1.8	0.0	0.0	0.0
PD3	75	1.2	0.0	0.0	13.3	1.3	0.0	0.0
PD4	107	13.6	1.2	0.0	66.4	27.1	0.0	0.0
*Memecylon*								
MU1	129	2.6	3.3	0.0	19.4	21.7	3.1	21.7
MU2	128	25.5	30.0	3.0	60.9	67.2	21.9	27.5
MU3	68	0.7	0.9	0.0	13.2	14.7	0.0	27.5
*Olea*								
OD1	106	0.0	0.3	0.0	0.9	1.9	0.0	Na
OD2	122	0.0	0.8	0.0	0.8	9.0	0.0	8.3
OD3	84	0.0	0.6	0.0	1.2	9.5	0.0	5.0
OD4	106	0.0	0.3	0.0	0.9	7.5	0.0	15.0
*Terminalia*								
TP1	42	1.5	3.1	0.0	9.5	11.9	0.0	0.0
TP2	40	0.0	0.0	0.0	0.0	0.0	0.0	0.0
TP3	45	10.6	20.6	0.1	37.8	48.9	4.4	0.0

## 4 Discussion

Air temperatures on land have increased, on average, by approximately 1.8°C since pre‐industrial times, and, by end of the century, are likely to exceed pre‐industrial temperatures by ~3.6°C. The overarching aim of our study is to shed some light on the question whether, in parts of the tropics, these increases may change which forest species will remain to be able to grow naturally, and, similarly, whether traditional agroforestry species like coffee and cocoa may still be grown in the parts of the world where they are currently being cultivated. We pursued this aim by examining one aspect which is whether the current and the future peak temperatures are already reaching temperature levels that are significantly affecting PSII/limiting performance (*T*_*max*_) of some tropical seasonally dry forest and agroforestry vegetation in India's Western Ghats, a very warm place.

Our continuous, long‐term leaf temperature data reveal that leaf temperatures of both forest and agroforestry species, during peak solar radiation, are regularly and substantially higher than air temperatures, frequently by up to 10°C–12°C, for most examined individuals (Figures 1 and S7). Measured leaf temperatures are among the highest ever reported in the literature. Thus, for the investigated species, leaf evaporative cooling is either not a protection mechanism used during this period of the year or is not effective for compensating heat gained by absorption of solar radiation. This is consistent with the findings of Still et al. (2022) that was based on data primarily from temperate latitude sites (where maximum leaf and air temperatures are substantially lower compared to our site), as well with Fauset et al. (2018) (Atlantic forest), Manzi et al. (2024), Kullberg et al. (2024) (tropical forest), while being inconsistent with the ‘homeothermy hypothesis’ of Michaletz et al. (2015). Maximum temperatures reached, one of the factors determining heat related damage to leaves, vary between leaves of a species as well as between species. This is also the case for measures of limits of performance (*T*_*max*_) and of damage to PSII (*T*_*5*_, *T*_*50*_), which vary between species within a range of 3°C–4°C. Among the four forest species, *Memecylon umbellatum* has the lowest *T*_*50*_ (47.8°C).

Leaf temperatures of all forest species surpass *T*_*max*_, the upper temperature limit below which CO_2_ can be assimilated, regularly. Typically, the time window during the day during which assimilation is not possible is on the order of minutes, that is, a limited period compared to daytime hours, thus, leaving time for carbon assimilation during the day. Therefore, by the criterion of exceedance of *T*_*max*_, the studied forest species are not expected to be seriously affected under current and future climate conditions. Threshold temperature measure (*T*_*50*_) associated with lasting damage are all higher than *T*_*max*_. For several of the forest species, *T*_*5*_, and for most of the agroforestry species, *T*_*5*_ and *T*_*50*_ are, nonetheless, regularly surpassed. In contrast, for the forest species, *T*_*50*_ is occasionally, although only rarely, surpassed (Figure 2; Table S5). This differs from earlier studies, such as Araújo et al. (2021) and Kitudom et al. (2022), which found that leaves were not, yet, exposed to temperatures above *T*_*50*_. It could be because, the climate at our site is particularly warm, or that records of these studies are short and do not cover the hottest period of the year.

Visual inspection of high‐temperature‐exposed leaves reveals some damage of leaves characteristic of excess heat for some of the species we investigated (Figure S5, Tables S5, and S6). Visual evidence of damage of a heat‐caused nature is particularly clear for agroforestry species (Figure S5). For the agroforestry species, on the one hand, there is, however, no clear correspondence between heat attributed damage and exceedance of thermal threshold measures *T*_*5*_ and *T*_*50*_, respectively (Table S6). On the other hand, there is a significant correlation between decrease of *F*_*v*_/*F*_*m*_ with percentage of leaves whose temperatures exceeded *T*_*50*_ and *T*_*5*_ (Figure S8). In conclusion for the agroforestry species, there is some, but not entirely clear‐cut, support for attributing visual damage to excess heat and a damaging effect on leaves when leaf temperatures exceed *T*_*50*_. These results suggest that additional factors may be contributing to vulnerability of these leaves under high temperatures which could for instance be related to access to water.

Consistent with the higher values of *T*_*50*_, we find substantially less visual evidence of heat related damage to leaves of forest species. For the four forest species, we do find some covariation between the exposure in units of time above threshold levels and the damage observed (Table 1). Specifically, the species for which there is some covariation is *Memecylon umbellatum* (for which *T*_*50*_ is the lowest among the four forest species).

An important factor for assessing the effect of leaf temperature threshold measures of leaf lethality is for how long the leaves are continuously exposed to temperatures higher than critical thresholds (e.g., Neuner and Buchner 2023). In this regard, it is important to note that *T*_*50*_, the measure of leaf lethality, is usually determined after an exposure of 30 min. Nevertheless, *T*_*50*_ is a measure of the effect of cumulative, not instantaneous, exposure above the threshold temperature. While we lack the data to determine the duration of exposure above thresholds for the agroforestry species, our long‐term records for the four forest species do permit us to assess relevant statistics for exposure durations above critical thresholds (Tables 1, and S5; Figure 4). These statistics reveal that the duration of uninterrupted exposure above threshold temperatures, for the forest species, occurs currently nearly never and for future air temperatures, very rarely when increased by 2°C and 4°C, assuming everything else is equal (e.g., that there is no deviation of 1‐to‐1 scaling of leaf and air temperature increases). *T*_*50*_ values of forest species are around 50°C (with exception of *Memecylon umbellatum*).

Given our results, a different picture of leaf heat damage under current and future climates emerges for agroforestry species versus forest species. Current and, thus, future land surface and air temperatures affect already negatively the studied agroforestry species in the region, and it may well be that they cannot be cultivated easily, possibly not at all, in the region in the future. Attribution to causes of leaf damage is somewhat ambiguous. Unfortunately, we do not have uninterrupted above threshold exposure statistics for the agroforestry species. For forest species, in contrast, the combination of higher threshold temperatures and the highly skewed nature of the distribution of uninterrupted exposure duration above threshold measures (Figure 4) likely mean that the leaves today, and for 2°C and 4°C future air temperature warming, may not be under lethal threat. The determinants of the highly skewed nature of this distribution are not clear, but could involve variation of leaf angles under varying wind directions and speed and, thus, variation in exposure to direct sunlight, or also changes in sensible heat exchange.

Overall, our results suggest a nuanced view of current and future threats to the Indian tropical forests and, possibly, to global tropical forests in already very warm areas, with regards to PSII vulnerability. First, the range between maximum leaf temperatures and the instantaneously interpreted, thresholds of PSII functioning is quite narrow and is increasingly closing in. Second, there is variation in these thresholds both between species as well as between individuals within a species. Third, in assessing deleterious effects using *T*_*5*_ and *T*_*50*_, it is important to be aware that *T*_*5*_ is a threshold indicative of onset of damage in contrast to *T*_*50*_, which is indicative of irreversible damage. Finally, because of the way *T*_*5*_ and *T*_*50*_ are determined, they are measures of damage caused by time of cumulated, above threshold exposure, and not instantaneous. Combining these considerations with our data, we find that many of the agroforestry species grown in the region may be difficult to cultivate in the long term in this region. One reason is that *T*_*50*_ values are lower than those of the forest species (Table S3A). A second reason, although somewhat speculative, is that most of these species grow in the understorey. It may also be related to exposure times, but for agroforestry species, we cannot test this with our data. Our conclusions for forest species differ. As mentioned, *T*_*50*_ values of forest trees tend to be higher than those of the agroforestry species. Furthermore, above threshold exposure durations of forest tree leaves tend to be much shorter than 30 min, today as well as for 2°C and 4°C temperature increase scenarios. Thus, most of the forest species may remain safe to irreversible leaf damage caused by temperature on PSII.

We note that we have determined *T*_*50*_ on dark adapted leaves. Krause et al. (2010) examined whether *T*_*50*_ measured on light exposed leaves were higher than that on dark adapted leaves and found that the former were approximately 1°C higher. If we apply such a correction, our conclusions from durations of exposure to the thresholds are strengthened. We further note that we have probed sun‐exposed, forest canopy leaves which will reach the highest temperatures of all canopy leaves. Shaded leaves will be less exposed to excessive temperatures and, thus, their photosystems at lesser heat exposure risk. Finally, we have determined *T*_*50*_ values post monsoon. *T*_*50*_ measured during the dry season are higher by approximately 1°C (Sastry and Barua 2017), which would further reduce our estimate of leaf damage risk. While exceedance of *T*_*50*_ is one important factor for future tropical forest resilience, this alone should not be the criterion to generalize temperature resilience of tropical forests. Although exceedance of upper thresholds like *T*_*50*_ might result in leaf necrosis and death which comes at a cost to the individual, connecting this to mortality will require other information, e.g., carbohydrate storage/reserve thtallows trees to regrow/flush leaves again after an extreme temperature event.

Our conclusions differ somewhat from the recent synthesis study of Doughty et al. (2023) regarding the future vulnerability of PSII. For the tropical forest we studied, we infer lesser vulnerability. There are several reasons. First, because *T*_*5*_ (or *T*_*crit*_ in Doughty et al. (2023) is an indicator of initiation of damage, while *T*_*50*_ is an indicator of irreversible damage, *T*_*50*_ is, in our view, a more appropriate measure to determine the true threshold of photosynthetic functioning and leaf damage. Second, different from Doughty et al. (2023), we do not find evidence for a nonlinear effect of ambient air temperature on leaf temperatures with leaves heating disproportionately with increasing ambient temperature (p. 107 of Doughty et al. 2023) because of temperature dependence of transpiration. Although our data cover quite a large range of ambient temperatures, leaf to air temperature differences do not increase with increasing air temperatures. This could be because, for our site, stomata are closed when leaf temperatures are highest in the day, irrespective of ambient temperatures (thus there cannot be “additional” downregulation of evapotranspirative cooling). Finally, and maybe most importantly, Doughty et al. (2023) did not take into consideration that *T*_*5*_ and *T*_*50*_ are measures of cumulative exposure, and not instantaneous, and our data show that it is important to take this into consideration.

Several other approaches have been used to assess the role played by high temperatures on tropical forest performance in different parts of the world. Locosselli et al. (2020) used tree rings to examine controls on tropical forest tree longevity in the tropics. They found that tropical forest tree longevity decreased above annual mean temperatures of 25.4°C while it did not depend on annual mean temperature at all below 25.4°C. Sullivan et al. (2020) used repeated forest censuses from widespread census networks in tropical South America, Africa and South‐East Asia, and a space for time substitution to determine sensitivity of forest productivity to high temperatures. They found that air temperature has a substantial effect on productivity and biomass stocks particularly in ‘the hottest forests (mean air temperatures > 32.2°C)’. Tropical biome wide, they did find substantial reductions in biomass stocks for a 2°C air temperature increase scenario. These studies point to factors other than PSII vulnerability, which contribute to decreases in tropical forest productivity in high‐temperature environments.

## 5 Summary and Conclusions

We investigated high‐temperature effects on health of tree leaves of tropical forest trees and agroforestry species in the Western Ghats of India, a very warm region, both by monitoring whether and how often leaf temperatures exceed physiological limits of PSII functioning and CO_2_ assimilation rates, as well as by determining whether exceedances of these limits lead to leaf damage indicators. The leaf temperatures are some of the highest observed in situ so‐far. We find, first, that there is a non‐negligible variation of thermal threshold measures both across species and individuals which needs to be taken into account when assessing PSII lethality. Post‐exposure diagnostics show evidence of PSII damage as well as visually determined heat related damage on a fraction of agroforestry leaves and, to a much lesser extent, on leaves of some of the forest species. However, attribution of damage to above threshold exposure remains ambiguous. Our data permit us to determine exposures of leaves to temperatures above *T*_*50*_ and durations of such continuous exposure events, both of which are critical in assessing lasting damage. This distribution is heavily skewed to short‐term exposure (much less than the 30 min exposure used to determine *T*_*50*_) both for current climate as well as for 2°C and 4°C air temperature increases (assuming everything else remain equal). Therefore, our results, altogether, suggest that based on PSII vulnerability measures alone, the forests at our very warm site should be resilient to warming. In contrast, agroforestry species are more vulnerable and thus their cultivation is very likely becoming more difficult in this region. Our site is located in a hot region of the tropics and leaf temperatures are reaching some of the highest leaf temperatures observed so far. Our results may, thus, have wider, somewhat optimistic, implications for tropical forest resilience under anthropogenic climate warming.

## Author Contributions

**Akhil Javad:** conceptualization, data curation, formal analysis, investigation, methodology, visualization, writing – review and editing. **Vikhyath Premugh:** investigation. **Rakesh Tiwari:** investigation. **Peddiraju Bandaru:** investigation. **Ron Sunny:** investigation. **Balachandra Hegde:** investigation, resources. **Santiago Clerici:** investigation, resources. **David Galbraith:** conceptualization, funding acquisition, methodology, project administration, supervision, writing – review and editing. **Manuel Gloor:** conceptualization, formal analysis, funding acquisition, methodology, project administration, supervision, visualization, writing – original draft, writing – review and editing. **Deepak Barua:** conceptualization, formal analysis, funding acquisition, methodology, project administration, supervision, visualization, writing – review and editing.

## Conflicts of Interest

The authors declare no conflicts of interest.

## Supporting information


**Data S1.** Supporting Information.

## Data Availability

The data that support the findings of this study are openly available in Dryad at https://doi.org/10.5061/dryad.tb2rbp0ch.
