# Supplementary material for: Leaf Temperatures in an Indian Tropical Forest Exceed Physiological Limits but Durations of Exposures Are Currently Not Sufficient to Cause Lasting Damage
Source: Glob Chang Biol. 2025 Feb 10;31(2):e70069. doi: 10.1111/gcb.70069 (PMC11808423; doi:10.1111/gcb.70069)
Supplement: Supplementary file 1 — Data S1. Supporting Information. [file GCB-31-e70069-s001.pdf]

Supplementary Information

Leaf temperatures in an Indian tropical forest exceed physiological limits but durations of exposures are currently not sufficient to cause lasting damage

A. Javad, V. Premugh, R. Tiwari, B. Peddiraju, R. Sunny, B. Hegde, S. Clerici, D. Galbraith, M. Gloor, D. Barua

Tables

Table S1 Agroforestry species names, growth habitats, light environments and origins.
Table S2 Physiological thresholds of forest and agroforestry species.
Table S3 A-D Summary and Post-hoc results of ANOVA on forest and agroforestry T_{50} values.
Table S4 A-B Summary and Post-hoc results of ANOVA on daily maximum leaf temperatures.
Table S5 A Percentages of days when daily maximum leaf temperatures exceeded the thresholds.
Table S5 B Average and maximum uninterrupted exposure of forest leaves to the thresholds.
Table S5 C Average and maximum uninterrupted exposure of forest leaves to the thresholds with acclimation.
Table S6 Percentages of agroforestry leaves with exposure to T_5 and T_{50} values.

Figures

Fig. S1 Location and Climatology of the tropical forest Site.
Fig. S2 Photographs of a thermistor pair attached on a leaf.
Fig. S3 Intercalibration of the handheld thermal camera.
Fig. S4 (A-B) F_v/F_m curves of Forest species (A) and Agroforestry species (B) used for estimation of T_{50}.
Fig. S5 Photographs of leaves with and without necrotic damages.
Fig. S6 (A-D) Time series of long-term leaf and air temperatures of the forest leaves.
Fig. S7 (A-D) Diurnal cycle and distributions of forest leaf-to-air temperature differences.
Fig. S8 Correlations between F_v/F_m and thermal safety margins, physiological thresholds and exposures.

Table S1 Species, family, common names, growth habitat, light environment, origin and native biomes of the agroforestry species used in the study. Origins of the species are taken from Plants of the World (POWO, 2024).

Species	Family	Common Name	Growth Habit	Light Environment	Origin	Native Biome of Non-Natives
Elettaria cardamomum (L.) Maton	*Zingiberaceae*	Cardamom	Geophyte	Understorey	Native	
Anacardium occidentale L.	*Anacardiaceae*	Cashew	Tree	Open	South America	Seasonally dry tropical biome
Manilkara zapota (L.) P. Royen	*Sapotaceae*	Chickoo	Tree	Open, semi-shade	Central and South America	Wet tropical biome
Cinnamomum verum J. Presl	*Lauraceae*	Cinnamon	Tree	Open, semi-shade	Sri Lanka	Wet tropical biome
Citrus L.	*Rutaceae*	Citrus	Tree	Open	Native	
Syzygium aromaticum (L.) Merr. & L.M.Perry	*Myrtaceae*	Clove	Tree/Shrub	Understorey	Maluku Islands	Wet tropical biome
Theobroma cacao L.	*Malvaceae*	Cocoa	Tree	Understorey	South America	Wet tropical biome
Coffea arabica L.	*Rubiaceae*	Coffee	Tree	Understorey	Central-East Africa	Seasonally dry tropical biome
Citrus × *limon* (L.) Osbeck	*Rutaceae*	Lemon	Tree	Semi-shade	Hybrid cultivar, non-native	
Piper nigrum L.	*Piperaceae*	Pepper	Vine	Understorey	Native	
Nephelium lappaceum L.	*Sapindaceae*	Rambutan	Tree	Open	Southeast Asia	Wet tropical biome
Syzygium samarangense (Blume) Merr. & L.M.Perry	*Myrtaceae*	Syzygium	Tree	Open	Native	
Vanilla planifolia Andrews	*Orchidaceae*	Vanilla	Epiphyte	Understorey	Central and South America	Wet tropical biome

Table S2 Photosynthetic thresholds – T_{opt} and T_{max} - of the forest species and damage based thresholds – T_5 and T_{50} – of the forest and the agroforestry species.

Species	Common Name	T_{opt} (^oC)	T_{max} (^oC)	$T_5 \pm SD$ (^oC)	$T_{50} \pm SD$ (^oC)
**Forestry Species**					
Psydrax dicoccos	*Psydrax*	28.27	43.37	46.43 ± 2.68	50.79 ± 2.25
Memecylon umbellatum	*Memecylon*	29.27	44.38	44.08 ± 1.52	47.81 ± 1.34
Olea dioica	*Olea*	31.37	46.35	43.95 ± 1.94	49.46 ± 1.33
Terminalia paniculata	*Terminalia*	32.17	45.50	44.22 ± 2.58	49.08 ± 2.10
**AgroForestry Species**					
Elettaria cardamomum	Cardamom			44.75 ± 1.10	48.57 ± 0.73
Anacardium occidentale	Cashew			45.36 ± 2.25	49.73 ± 1.65
Manilkara zapota	Chickoo			42.14 ± 2.24	46.70 ± 1.81
Cinnamomum verum	Cinnamon			46.06 ± 1.84	48.49 ± 1.69
Citrus	Citrus			45.63 ± 2.63	48.62 ± 2.26
Syzygium aromaticum	Clove			43.98 ± 2.37	48.23 ± 1.87
Theobroma cacao	Cocoa			43.93 ± 2.35	46.03 ± 1.90
Coffea arabica	Coffee			44.51 ± 1.42	49.05 ± 1.17
Citrus limon	Lemon			45.50 ± 1.05	47.03 ± 0.92
Piper nigrum	Pepper			44.16 ± 1.52	47.77 ± 1.37
Nephelium lappaceum	Rambutan			45.50 ± 1.78	49.16 ± 1.48
Syzygium samarangense	Syzygium			46.44 ± 1.57	48.01 ± 1.32
Vanilla planifolia	Vanilla			42.57 ± 1.77	48.95 ± 1.45

Table S3 A-D Summary of ANOVA tests done on T_{50} values to test the effects of Forest versus Agroforestry (A), Species (B) and light environment (D). (C) contains the Post-hoc Tukey results of the ANOVA from (B). `Type`: Forest versus Agroforestry. `Environment`: Understorey versus Semi-shade vs Open as in Table S1.

A

```
              Df Sum Sq Mean Sq F value   Pr(>F)
Type           1  25.83  25.826  25.183 2.11e-06 ***
Type:Species  15 135.18   9.012   8.788 8.04e-13 ***
Residuals    106 108.71   1.026
---
Signif. codes:  0 ‘***’ 0.001 ‘**’ 0.01 ‘*’ 0.05 ‘.’ 0.1 ‘ ’ 1
```

B

```
             Df Sum Sq Mean Sq F value   Pr(>F)
Species      16  161.0  10.063   9.812 1.38e-14 ***
Residuals   106  108.7   1.026
---
Signif. codes:  0 ‘***’ 0.001 ‘**’ 0.01 ‘*’ 0.05 ‘.’ 0.1 ‘ ’ 1
```

C

**Species**	**a**	**b**	**c**	**d**	**e**	**f**
Canthium	a					
Cashew	a	b				
Olea	a	b	c			
Rambutan	a	b	c	d		
Coffee	a	b	c	d		
Terminalia	a	b	c	d		
Vanilla	a	b	c	d		
Citrus		b	c	d	e	
Cardamom		b	c	d	e	
Clove		b	c	d	e	
Cinnamon		b	c	d	e	
Syzygium			c	d	e	f
Pepper			c	d	e	f
Memecylon			c	d	e	f
Lemon				d	e	f
Chickoo					e	f
Cocoa						f

D

```
                     Df Sum Sq Mean Sq F value   Pr(>F)
Environment           2  47.35  23.673  23.084 4.76e-09 ***
Environment:Species  14 113.66   8.118   7.916 3.02e-11 ***
Residuals           106 108.71   1.026
---
Signif. codes:  0 ‘***’ 0.001 ‘**’ 0.01 ‘*’ 0.05 ‘.’ 0.1 ‘ ’ 1
```

Table S4 A-B (A) ANOVA results of daily maximum leaf temperatures (Fig. 2). One-way ANOVA was done across species with individuals of each species as a nested variable. (B) Post-hoc Tukey results.

A

```
                    Df Sum Sq Mean Sq F value Pr(>F)
Species              3   3149  1049.5   78.96 <2e-16 ***
Species:Individual  10   8400   840.0   63.20 <2e-16 ***
Residuals         1262  16774    13.3
---
Signif. codes:  0 ‘***’ 0.001 ‘**’ 0.01 ‘*’ 0.05 ‘.’ 0.1 ‘ ’ 1
```

B

**Species**	**Mean Daily Maximum Leaf Temperature (^oC)**	**SE (^oC)**	**a**	**b**
Olea	38.17	0.01	a	
Psydrax	38.59	0.01	a	
Terminalia	39.09	0.04	a	
Memecylon	42.02	0.01		b

Table S5 A Percentages of days during the measurement period where daily maximum leaf temperatures exceeded the physiological thresholds - T_{max}, T_5, and T_{50} - for leaves of the four forest species investigated in the study (Table 1, Fig. 2, Fig. S5) under current climate (0^oC), and, $+2^oC$ and $+4^oC$ warming scenarios. Z-score of each leaf shows how skewed the distribution of leaf-to-air temperature differences are from zero (Fig. S6 A-D).

		Percentage of Days		
**Species/ Individual**	**Z score (-)**	$T_{leaf} > T_{max}$− $0^oC/2^oC/4^oC$ (%)	$T_{leaf} > T_5$− $0^oC/2^oC/4^oC$ (%)	$T_{leaf} > T_{50}$− $0^oC/2^oC/4^oC$ (%)
Psydrax				
PD1	0.14	2.7/ 6.3/10.7	0.0/ 0.0/ 3.6	0.0/ 0.0/ 0.0
PD2	0.32	1.8/ 4.5/14.3	0.0/ 0.0/ 2.7	0.0/ 0.0/ 0.0
PD3	0.35	13.3/28.0/34.7	1.3/ 4.0/20.0	0.0/ 0.0/ 1.3
PD4	0.46	66.4/85.0/92.5	27.1/56.1/78.5	0.0/ 1.9/24.3
Memecylon				
MU1	0.64	19.4/38.8/52.7	21.7/40.3/55.0	3.1/10.1/26.4
MU2	0.90	60.9/80.5/85.9	67.2/82.0/87.5	21.9/46.9/68.8
MU3	0.50	13.2/25.0/44.1	14.7/30.9/44.1	0.0/ 5.9/16.2
Olea				
OD1	1.10	0.9/ 1.9/ 5.7	1.9/ 8.5/16.0	0.0/ 0.0/ 0.9
OD2	0.10	0.8/ 6.6/19.7	9.0/23.0/39.3	0.0/ 0.0/ 4.1
OD3	0.25	1.2/ 8.3/15.5	9.5/16.7/23.8	0.0/ 1.2/ 2.4
OD4	0.38	0.9/ 4.7/13.2	7.5/15.1/23.6	0.0/ 0.0/ 2.8
Terminalia				
TP1	0.33	9.5/11.9/21.4	11.9/19.0/21.4	0.0/ 4.8/ 9.5
TP2	0.62	0.0/ 0.0/ 2.5	0.0/ 2.5/ 5.0	0.0/ 0.0/ 0.0
TP3	0.84	37.8/53.3/71.1	48.9/60.0/73.3	4.4/22.2/40.0

Table S5 B Mean and maximum uninterrupted leaf temperature exposure to physiological thresholds - T_{max}, T_5, and T_{50} - across the measurement period for the leaves of the 4 forest species (Table 1, Fig. S5) under current climate (^oC), and, +2^oC and +4^oC warming scenarios.

	Mean Exposure			Maximum Exposure		
**Species/ Individual**	**$T_{leaf} > T_{max}-$ 0^oC/ 2^oC/ 4^oC (min/day)**	**$T_{leaf} > T_5-$ 0^oC/ 2^oC/ 4^oC (min/day)**	**$T_{leaf} > T_{50}-$ 0^oC/ 2^oC/ 4^oC (min/day)**	**$T_{leaf} > T_{max}-$ 0^oC/2^oC/4^oC (min)**	**$T_{leaf} > T_5-$ 0^oC/2^oC/4^oC (min)**	**$T_{leaf} > T_{50}-$ 0^oC/2^oC/4^oC (min)**
Psydrax						
PD1	0.0/ 0.7/ 2.6	0.0/ 0.0/ 0.2	0.0/ 0.0/ 0.0	1/ 17/ 48	0/ 0/ 11	0/ 0/ 0
PD2	0.1/ 0.7/ 3.2	0.0/ 0.0/ 0.3	0.0/ 0.0/ 0.0	2/ 6/ 31	0/ 0/ 4	0/ 0/ 0
PD3	1.2/ 4.6/16.2	0.0/ 0.6/ 2.2	0.0/ 0.0/ 0.0	12/ 43/ 43	3/ 7/ 22	0/ 0/ 3
PD4	13.6/36.0/70.9	1.2/ 7.0/ 22.4	0.0/ 0.0/ 0.8	21/ 62/129	6/ 19/ 44	0/ 2/ 5
Memecylon						
MU1	2.6/ 9.8/25.9	3.3/11.8/ 28.6	0.0/ 0.7/ 3.8	11/ 50/120	11/ 60/120	1/ 5/ 11
MU2	25.5/58.6/95.0	30.0/64.7/100.0	3.0/12.1/34.1	130/180/180	130/180/210	30/110/130
MU3	0.7/ 4.9/13.9	0.9/ 5.8/ 16.0	0.0/ 0.2/ 1.2	4/ 13/ 85	5/ 19/ 85	0/ 2/ 5
Olea						
OD1	0.0/ 0.2/ 0.8	0.3/ 1.0/ 3.0	0.0/ 0.0/ 0.1	3/ 6/ 13	6/ 13/ 31	0/ 3/ 4
OD2	0.0/ 0.5/ 3.4	0.8/ 4.4/ 11.8	0.0/ 0.0/ 0.1	1/ 3/ 10	4/ 16/ 77	0/ 0/ 2
OD3	0.0/ 0.4/ 2.2	0.6/ 2.8/ 7.8	0.0/ 0.0/ 0.0	1/ 3/ 9	5/ 9/ 19	0/ 1/ 1
OD4	0.0/ 0.1/ 1.9	0.3/ 2.3/ 6.3	0.0/ 0.0/ 0.0	1/ 1/ 8	3/ 12/ 20	0/ 0/ 1
Terminalia						
TP1	1.5/ 4.2/ 9.5	3.1/ 7.5/ 14.4	0.0/ 0.2/ 1.9	4/ 32/ 51	24/ 50/ 89	0/ 2/ 10
TP2	0.0/ 0.0/ 0.3	0.0/ 0.2/ 1.1	0.0/ 0.0/ 0.0	0/ 0/ 12	0/ 3/ 13	0/ 0/ 0
TP3	10.6/28.2/54.6	20.6/43.4/ 75.2	0.1/ 2.9/13.5	12/ 92/175	51/130/206	1/ 5/ 19

Table S5 C Mean and maximum uninterrupted exposure duration to physiological threshold - T_{50} - across the measurement period for the leaves of the 4 forest species under current climate (^oC), and, $+2^oC$ and $+4^oC$ warming scenarios. Here, T_{50} values are assumed to have acclimated at a rate of 0.38°C per 1°C rise in air temperatures.

**Species/ Individual**	**Mean Exposure $T_{leaf} > T_{50}-$ $0^oC/\ 2^oC/\ 4^oC$ (min/day)**	**Maximum Exposure $T_{leaf} > T_{50}-$ $0^oC/\ 2^oC/\ 4^oC$ (min)**
Psydrax		
PD1	0.0/0.0/ 0.0	0/ 0/ 0
PD2	0.0/0.0/ 0.0	0/ 0/ 0
PD3	0.0/0.0/ 0.0	0/ 0/ 0
PD4	0.0/0.0/ 0.1	0/ 2/ 2
Memecylon		
MU1	0.0/0.3/ 1.4	1/ 4/ 10
MU2	3.0/7.8/16.6	30/110/110
MU3	0.0/0.0/ 0.3	0/ 1/ 3
Olea		
OD1	0.0/0.0/ 0.0	0/ 0/ 3
OD2	0.0/0.0/ 0.0	0/ 0/ 0
OD3	0.0/0.0/ 0.0	0/ 0/ 1
OD4	0.0/0.0/ 0.0	0/ 0/ 0
Terminalia		
TP2	0.0/0.1/ 0.7	0/ 2/ 3
TP3	0.0/0.0/ 0.0	0/ 0/ 0
TP3	0.1/1.2/ 5.2	1/ 4/ 6

Table S6 Percentages of top-canopy, sun-exposed leaves of agroforestry species with leaf temperatures measured during the peak solar irradiation (Fig. 4) exceeded physiological thresholds - T_5 and T_{50}. Percentages of sun-exposed leaves with some form of necrotic damage that we attribute to excess heat (Fig. S5) are also given.

**Species**	**Exposure** $T_{leaf} > T_5$ **(% of leaves)**	**Exposure** $T_{leaf} > T_{50}$ **(% of leaves)**	**Visual Heat Damage (%)**
Cardamom	9.3	0.0	15.3
Cashew	0.0	0.0	5.4
Chickoo	18.6	1.2	8.8
Cinnamon	5.8	1.9	NA
Citrus	5.3	0.0	3.3
Clove	0.0	0.0	15.6
Cocoa	61.5	23.1	13.5
Coffee	0.0	0.0	21.7
Lemon	34.8	13.0	8.3
Pepper	2.8	0.0	15.1
Rambutan	17.2	1.6	42.8
Syzygium	0.0	0.0	10.3
Vanilla	25.0	0.0	7.2

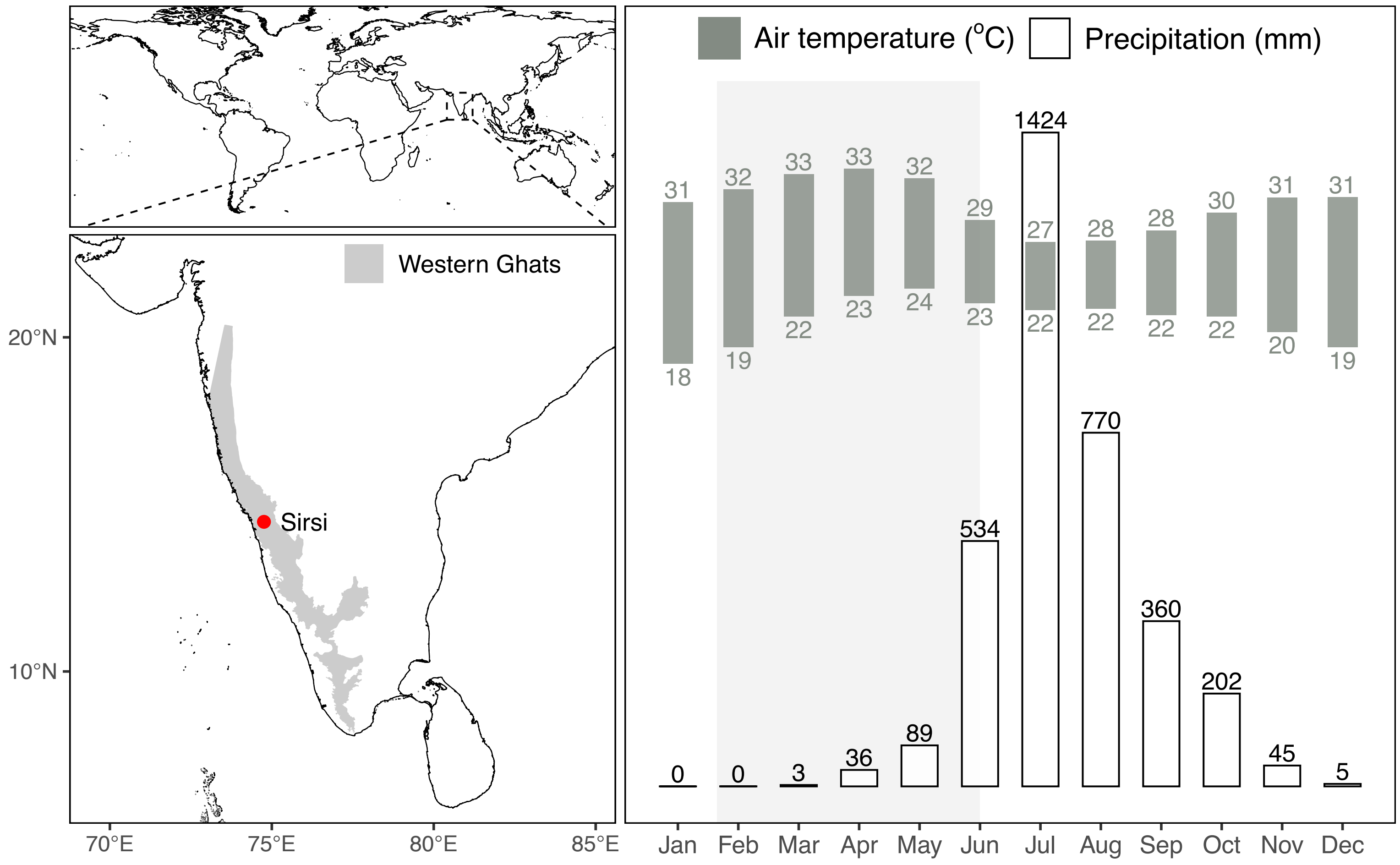

Fig. S1 Location of the measurement site and climatology of the seasonal cycle of precipitation and air temperature made using data from Climate Research Unit (CRU TS v4.08, Harris et al., 2020).

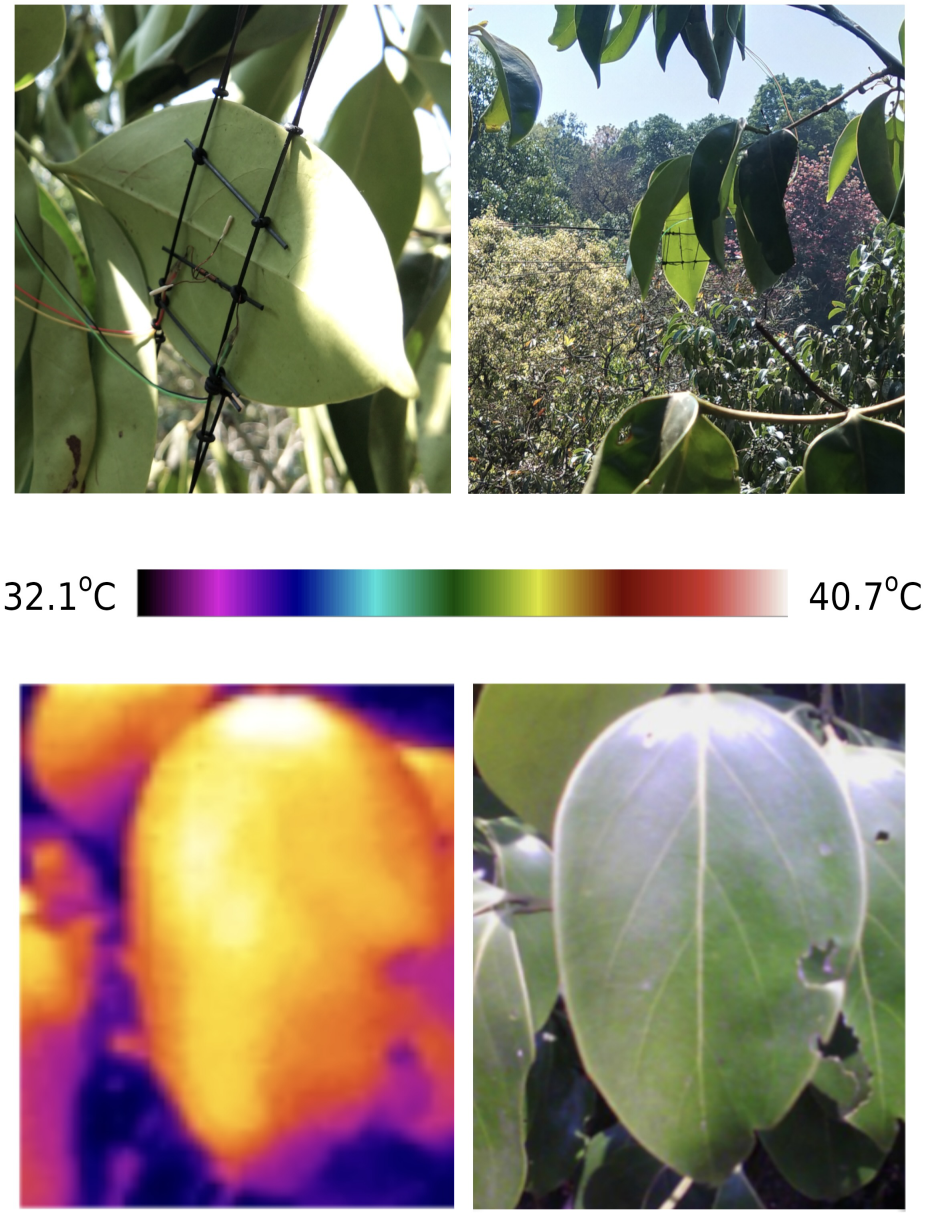

Fig. S2 Example of the installation of a first thermistor touching leaf surface from below and a second thermistor being bent downwards to measure air temperature adjacent to the leaf (upper panels) and example of thermal and visible image measured with handheld thermal camera (FLIR C2) (lower panels).

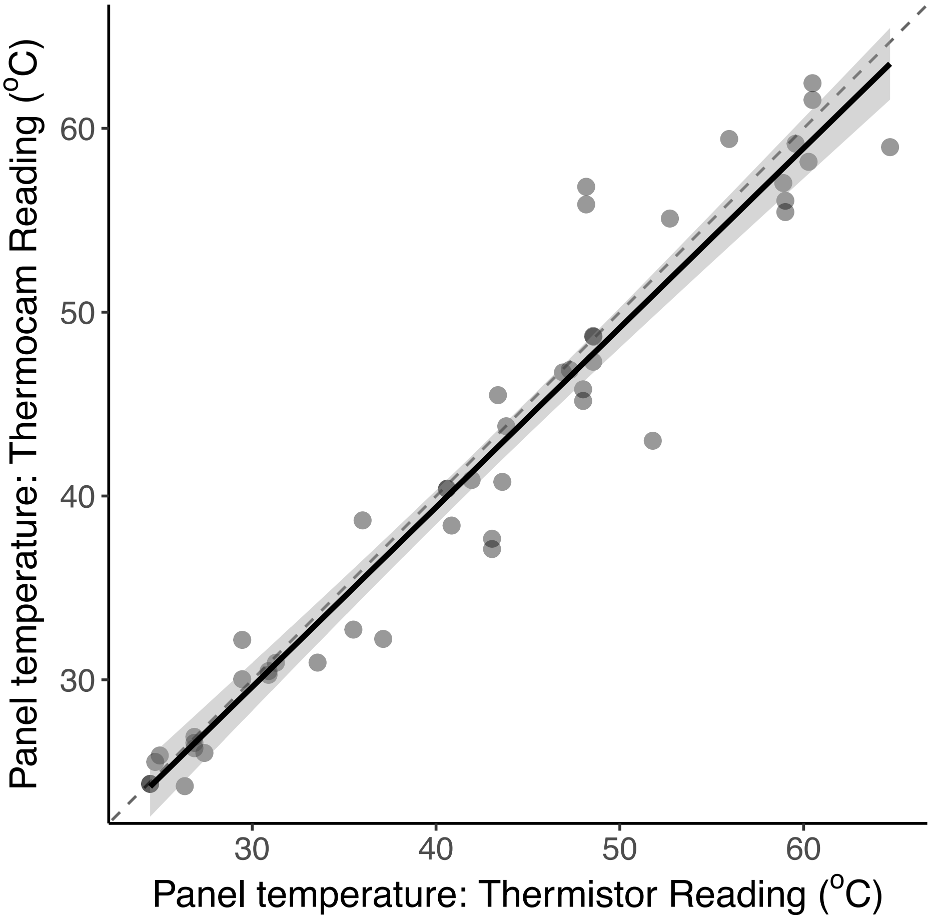

Fig. S3 Comparison of panel temperatures measured at the same time with thermistor and handheld thermal camera.

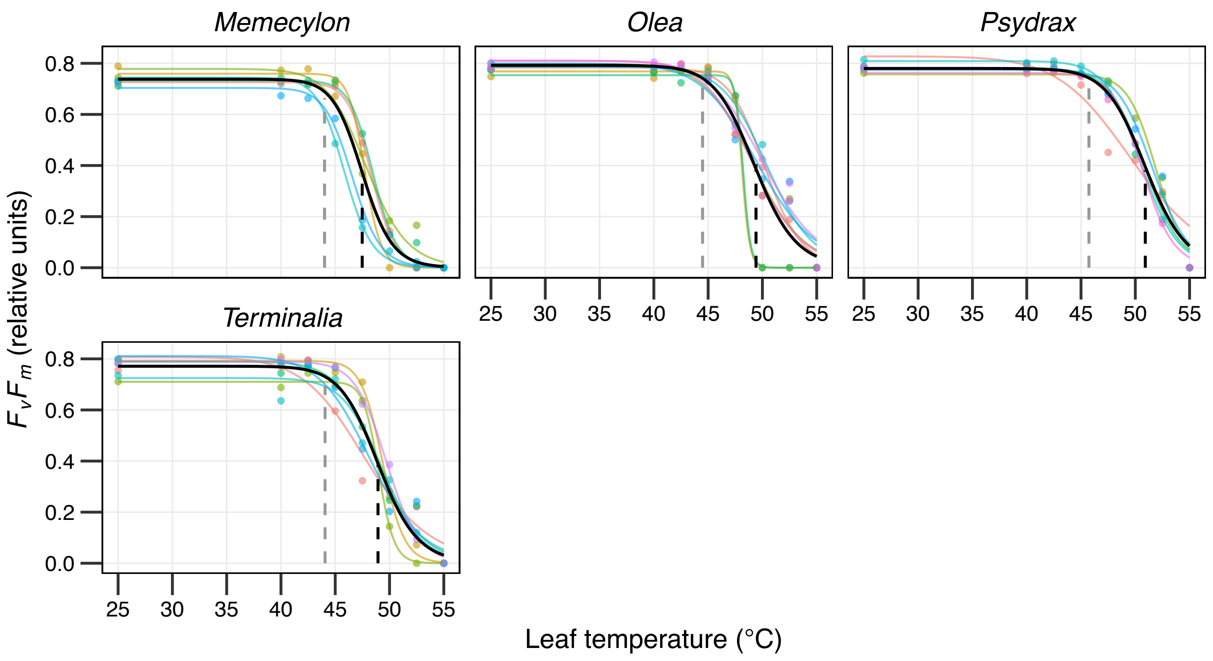

Fig. S4 A Dark-adapted, quantum efficiency (F_v/F_m) of PSII measured at different leaf temperatures to estimate T_5 and T_{50}. Figure shows the fitted curves for the four forest species. Coloured points and lines represent measured data and fitted curves, respectively, for different individuals of a given species. Average T_5, and T_{50} across individuals of each species are marked using grey and black dashed lines respectively.

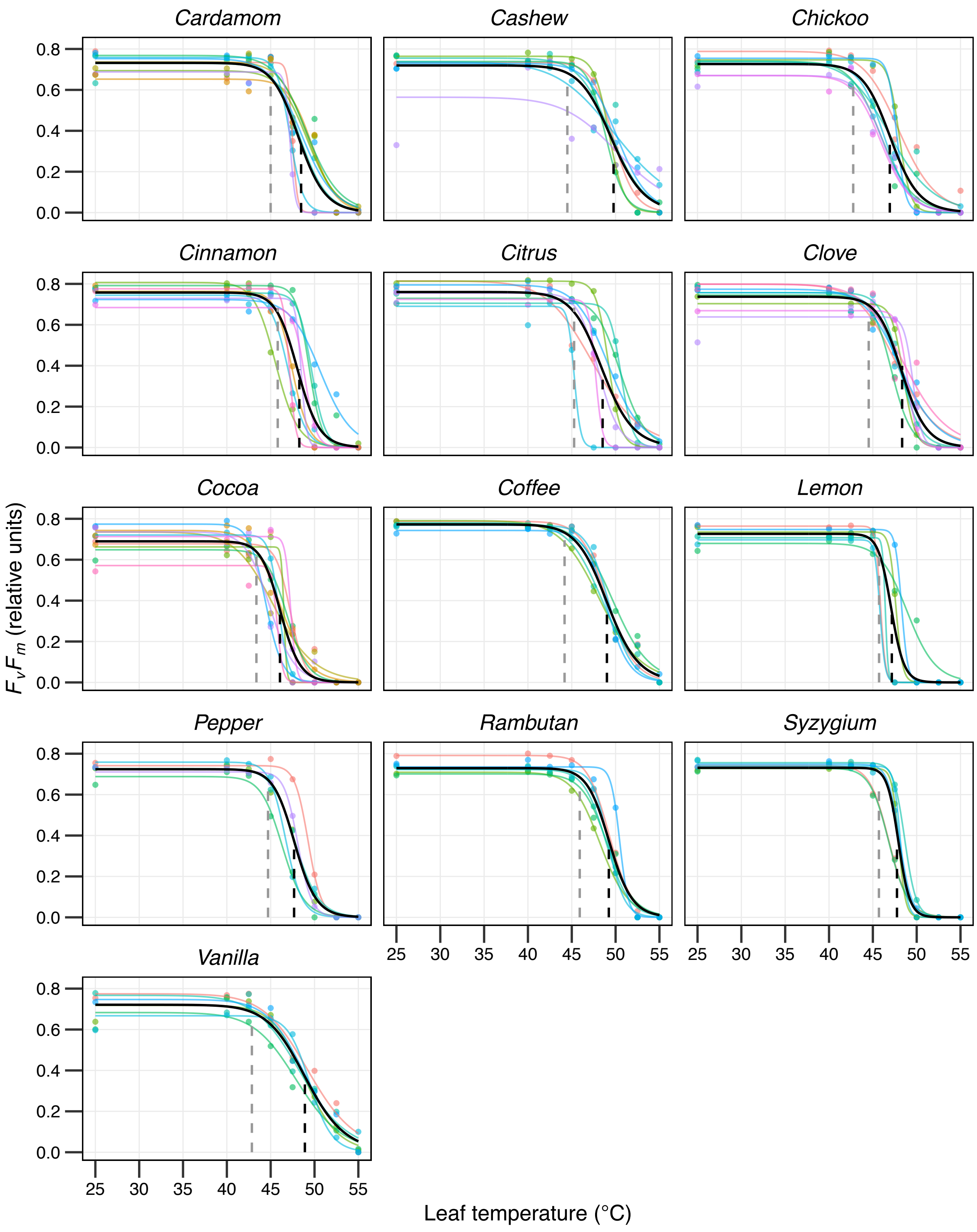

Fig. S4 B Dark-adapted, quantum efficiency (F_v/F_m) of PSII measured at different leaf temperatures to estimate T_5 and T_{50}. Figure shows the fitted curves for the 13 agroforestry species in the study. Coloured lines and points represent fitted curves and measured data, respectively, for different individuals of a given species. Average T_5, and T_{50} across individuals of each species are marked using grey and black dashed lines respectively.

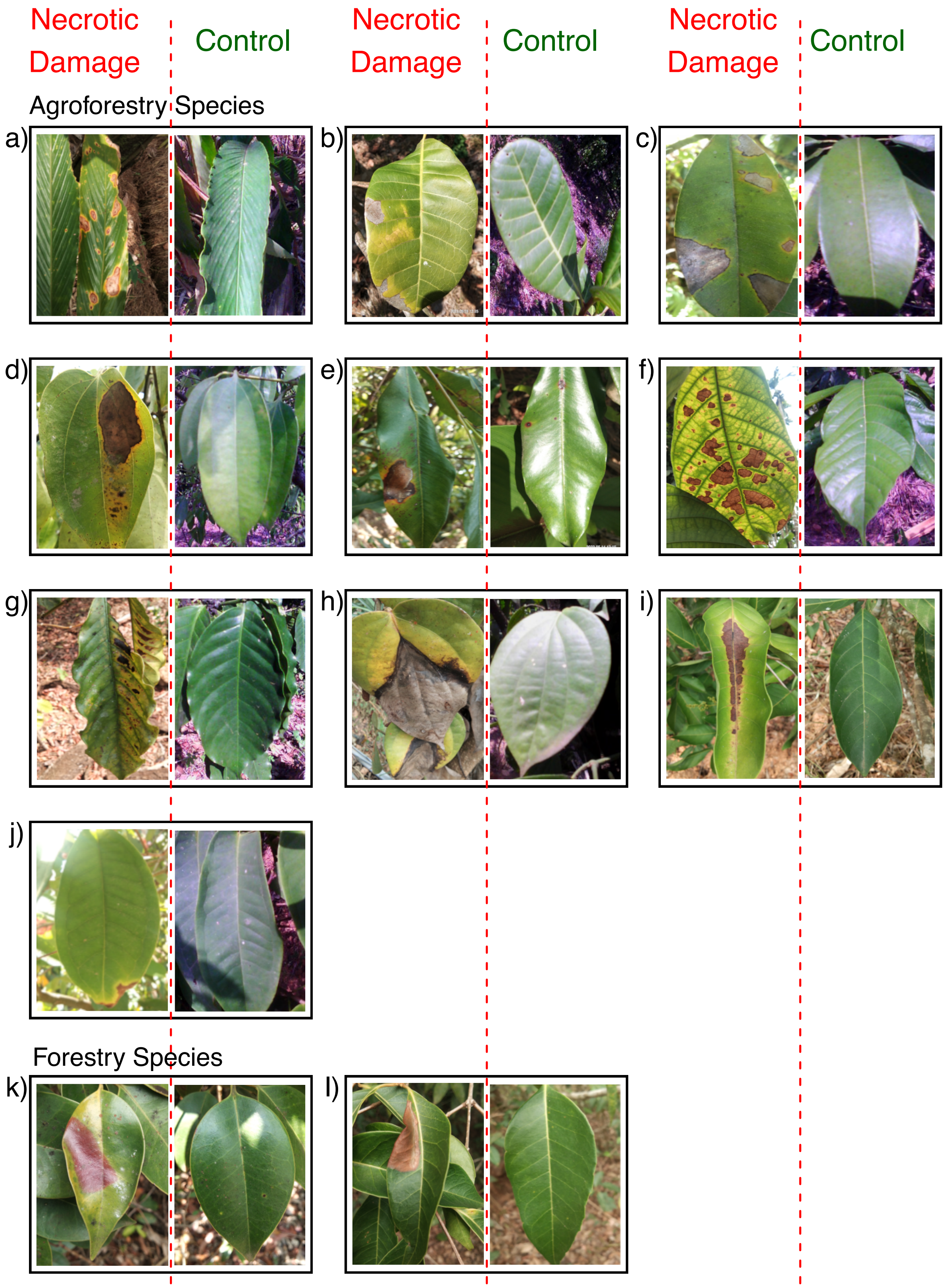

Fig. S5 Photographs of leaves made at the end of the measurement period (end of dry season) with and without visible damage we attribute to excess heat (as opposed e.g. to damage caused by a fungal disease). a. Cardamom, b. Cashew, c. Chickoo, d. Cinnamon, e. Clove, f. Cocoa, g. Coffee, h. Pepper, i. Rambutan, j. Syzygium, k. *Memecylon*, l. *Olea*

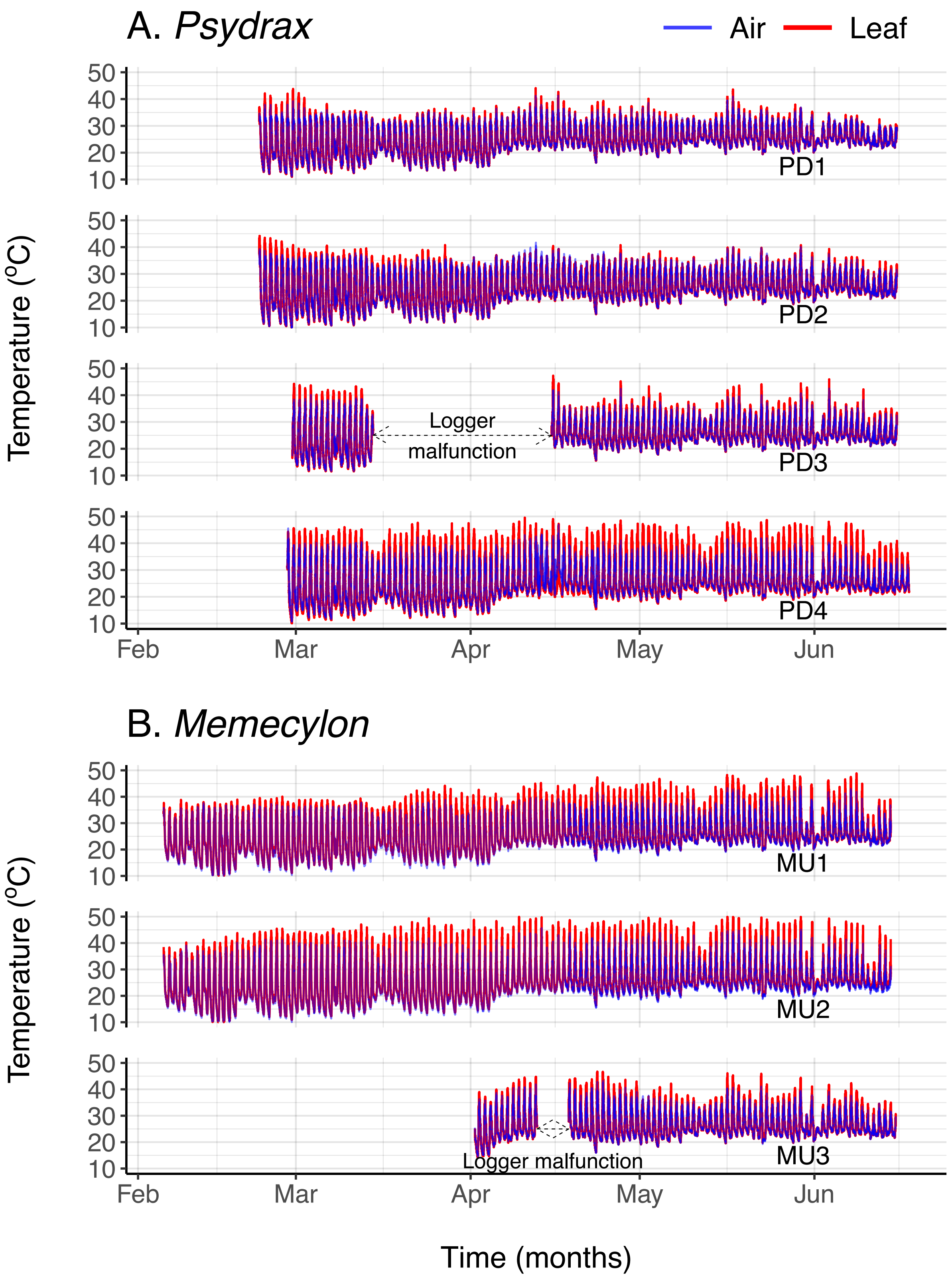

Fig. S6 A-B Leaf and below leaf air temperature time-series of two forest species probed in this study - *Canthium* (A) and *Memecylon* (B). The temperatures were measured using thermistors installed as shown in Fig. S2. Leaves of a species probed are from different individuals except MU2.

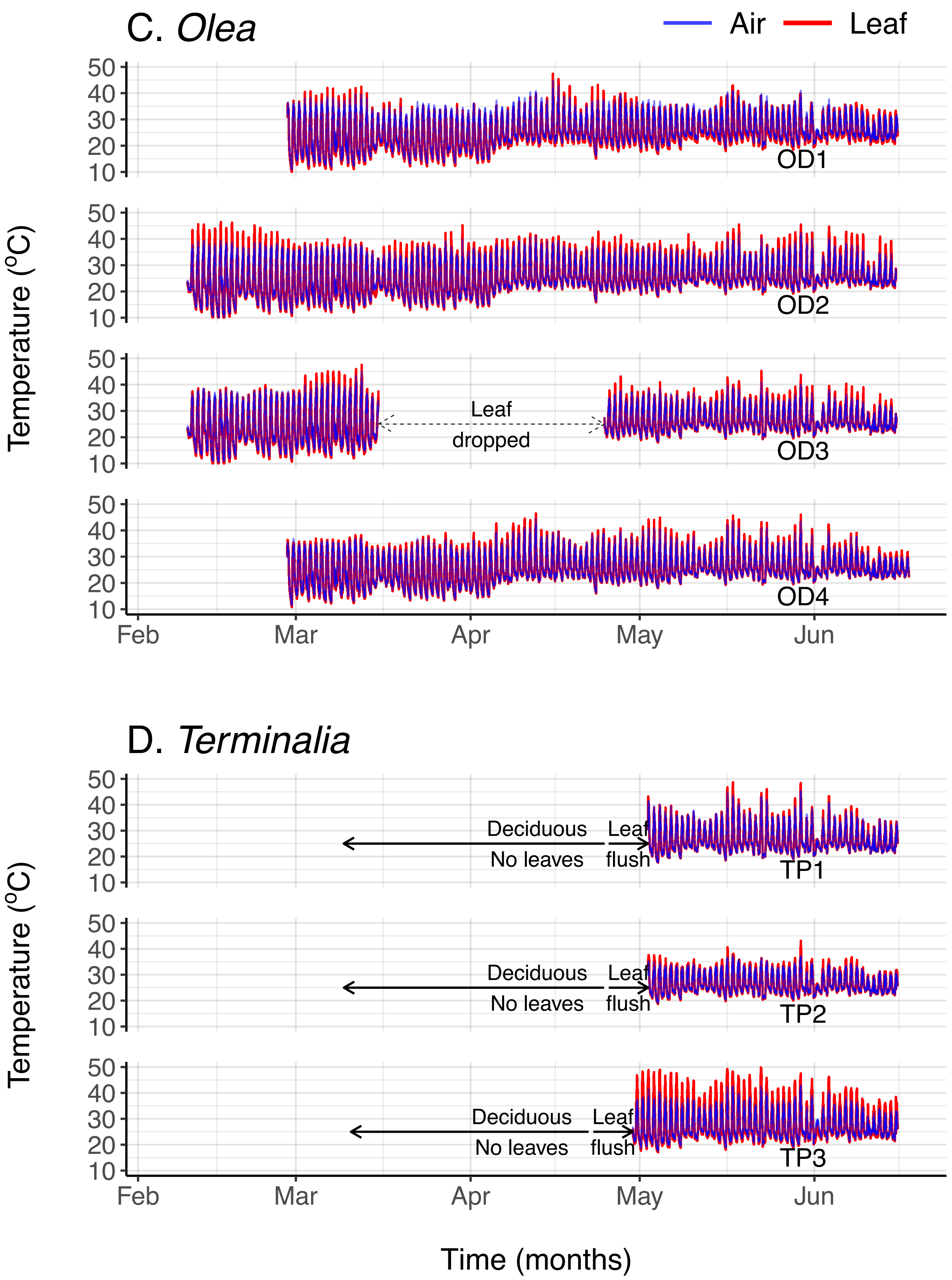

Fig. S6 C-D Leaf and below leaf air temperature time-series of two forest species probed in this study - *Olea* (C) and *Terminalia* (D). The temperatures were measured using thermistors installed as shown in Fig. S2. Leaves of a species probed are from different individuals.

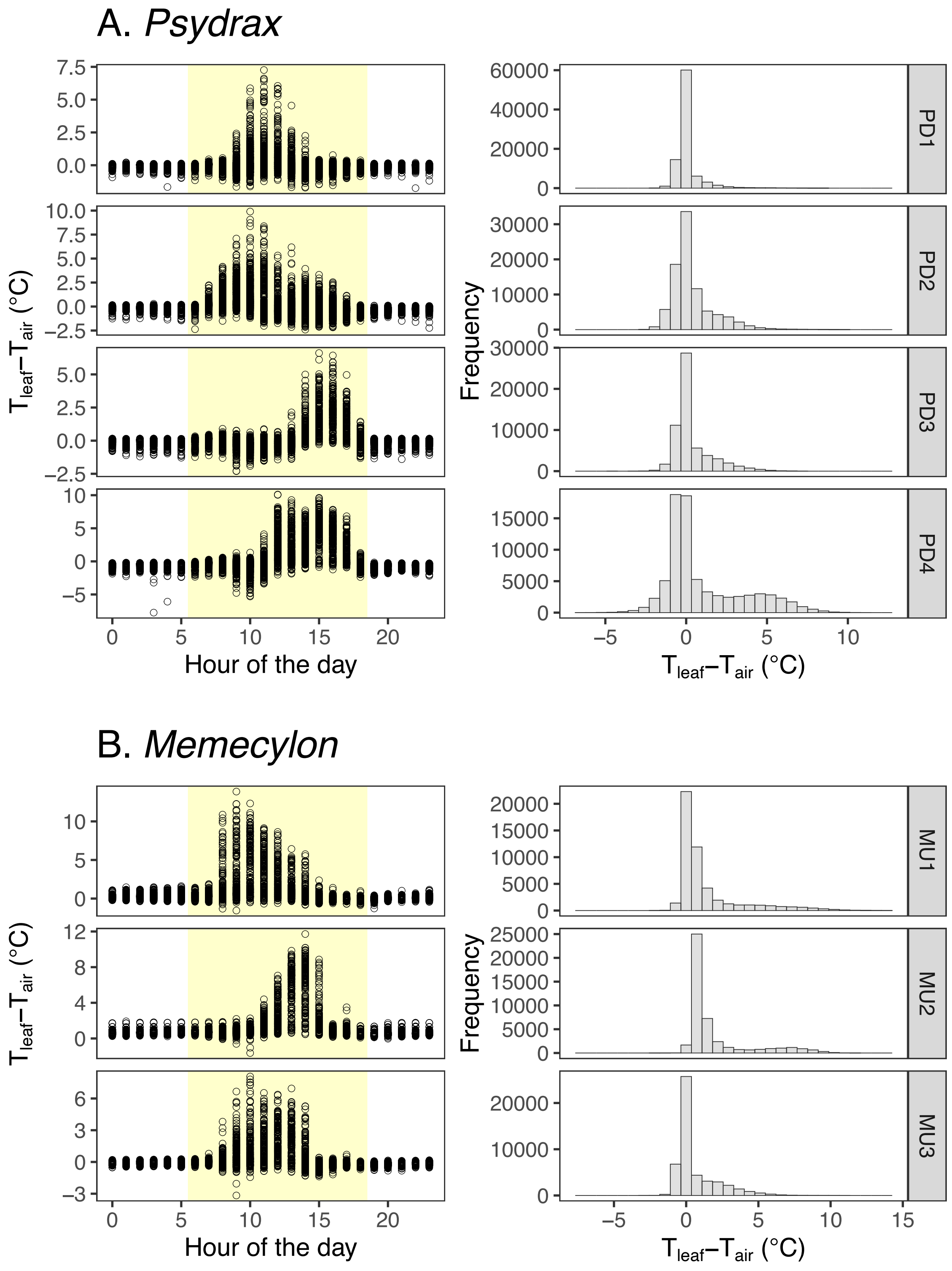

Fig. S7 A-B Daily cycle and histogram of leaf-air temperature difference measured with thermistors on sun-exposed leaves of two four forest species probed in this study - *Canthium* (A) and *Memecylon* (B). Leaves of a species probed are from different individuals except MU2.

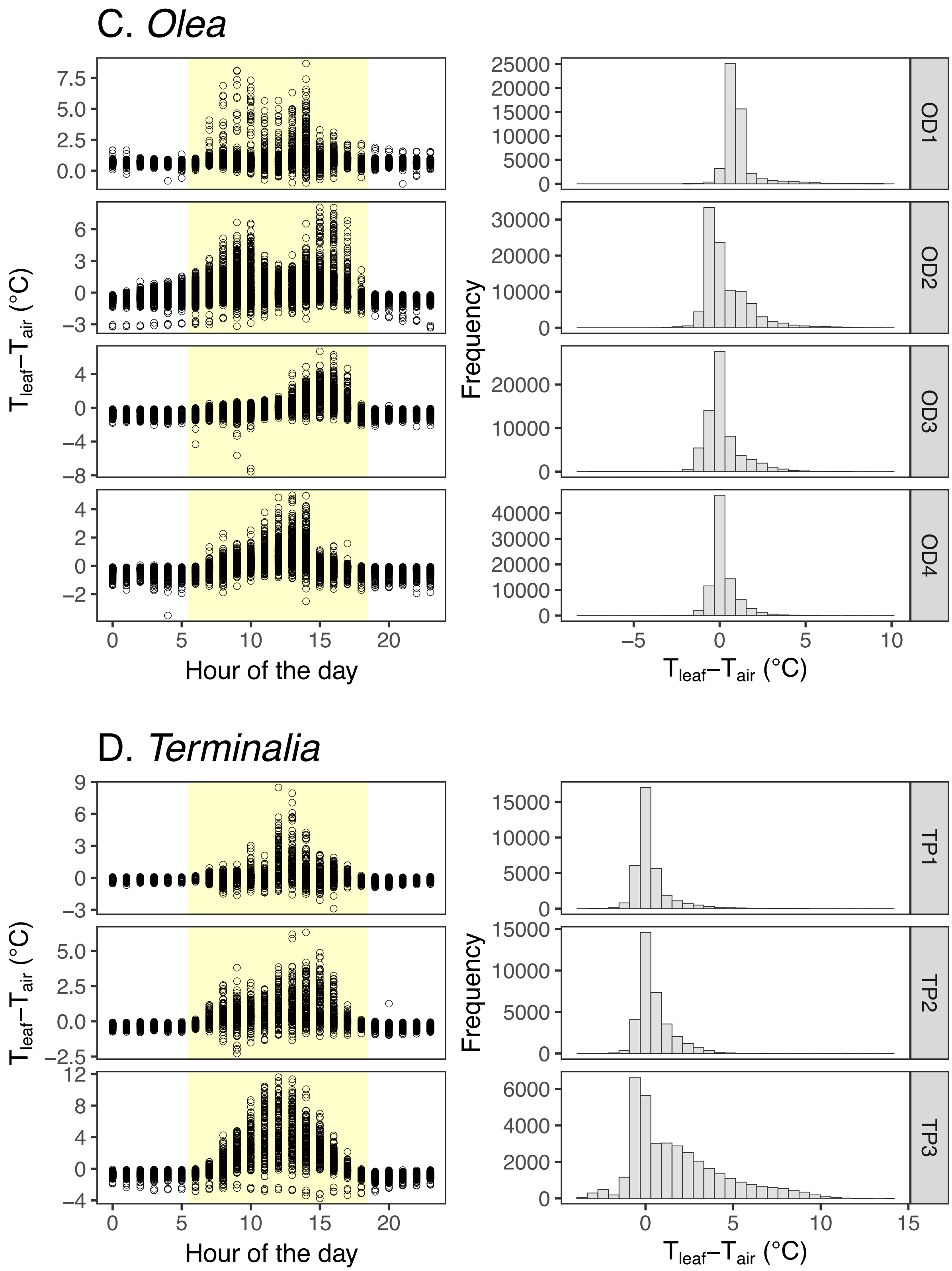

Fig. S7 C-D Daily cycle and histogram of leaf-air temperature difference measured with thermistors on sun-exposed leaves of two four forest species probed in this study - *Olea* (C) and *Terminalia* (D). Leaves of a species probed are from different individuals.

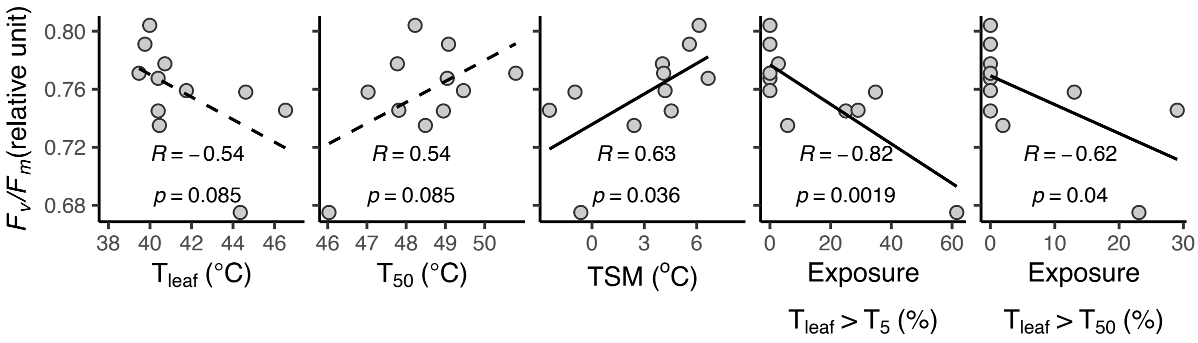

Fig. S8 Correlations between F_v/F_m and foliar traits, thermal safety margin ($T_{50} - T_{leaf}$), and percentage of leaves whose temperatures exceeded T_5 and T_50.
